# Renewable Hydrocarbon Production from Waste Cottonseed Oil Pyrolysis and Catalytic Upgrading of Vapors with Mo-Co and Mo-Ni Catalysts Supported on γ-Al_2_O_3_

**DOI:** 10.3390/nano11071659

**Published:** 2021-06-24

**Authors:** Josué Alves Melo, Mirele Santana de Sá, Ainara Moral, Fernando Bimbela, Luis M. Gandía, Alberto Wisniewski

**Affiliations:** 1 Petroleum and Energy from Biomass Research Group (PEB), Federal University of Sergipe, UFS, São Cristóvão 49100 000, SE, Brazil; josuecedro@hotmail.com (J.A.M.); mirelesantana@live.com (M.S.d.S.); 2 Grupo de Reactores Químicos y Procesos para la Valorización de Recursos Renovables, Institute for Advanced Materials and Mathematics (INAMAT2), Universidad Pública de Navarra (UPNA), 31006 Pamplona, Spain; moral.ainara@gmail.com (A.M.); fernando.bimbela@unavarra.es (F.B.); lgandia@unavarra.es (L.M.G.)

**Keywords:** biofuels, vegetable oil, pyrolysis, deoxygenation, decarboxylation, molybdenum, biomass, triacylglycerols

## Abstract

In this work, the production of renewable hydrocarbons was explored by the means of waste cottonseed oil (WCSO) micropyrolysis at 500 °C. Catalytic upgrading of the pyrolysis vapors was studied using α-Al_2_O_3_, γ-Al_2_O_3,_ Mo-Co/γ-Al_2_O_3_, and Mo-Ni/γ-Al_2_O_3_ catalysts. The oxygen removal efficiency was much lower in non-catalytic pyrolysis (18.0%), whilst γ-Al_2_O_3_ yielded a very high oxygen removal efficiency (91.8%), similar to that obtained with Mo-Co/γ-Al_2_O_3_ (92.8%) and higher than that attained with Mo-Ni/γ-Al_2_O_3_ (82.0%). Higher conversion yields into total renewable hydrocarbons were obtained with Mo-Co/γ-Al_2_O_3_ (61.9 wt.%) in comparison to Mo-Ni/γ-Al_2_O_3_ (46.6%). GC/MS analyses showed a relative chemical composition of 31.3, 86.4, and 92.6% of total renewable hydrocarbons and 58.7, 7.2, and 4.2% of oxygenated compounds for non-catalytic bio-oil (BOWCSO), BOMoNi and BOMoCo, respectively. The renewable hydrocarbons that were derived from BOMoNi and BOMoCo were mainly composed by olefins (35.3 and 33.4%), aromatics (31.4 and 28.9%), and paraffins (13.8 and 25.7%). The results revealed the catalysts’ effectiveness in FFA decarbonylation and decarboxylation, as evidenced by significant changes in the van Krevelen space, with the lowest O/C ratio values for BOMoCo and BOMoNi (O/C = 0–0.10) in relation to the BOWCSO (O/C = 0.10–0.20), and by a decrease in the presence of oxygenated compounds in the catalytic bio-oils.

## 1. Introduction

Current forecasts indicate that air traffic volumes will double in the next 15 years. By 2034, air passenger traffic and air freight traffic are both expected to be more than doubled when compared to 2016 [1]. In fact, despite the recent decay in air traffic caused by the COVID-19 pandemic, the most recent forecasts indicate that only a 4% downward adjustment of pre-COVID forecasts will take place, and it is expected that the total global fleet will reach numbers ranging between 45,000 and 48,000 airplane units by 2038–2039 [2]. Allied to the growth of the aviation sector is the increase of the consumption of petroleum-based kerosene, which is also called fossil jet fuel (Jet A1) and emissions of greenhouse gases (GHG) [3,4]. Currently, the aviation sector is estimated to contribute with 2.0–3.5% of global CO_2_ emissions into the atmosphere [5,6] and, as result of the growth, the aviation’s contribution to global fossil fuel CO_2_ emissions could grow to 4.6–20.2% by mid-century [7].

Because of the increasing consumption of Jet A1 and the environmental impact, the Carbon Offset and Reduction Scheme for International Aviation (CORSIA) was IATA’s primary focus in 2017, which have a carbon-neutral growth from 2020 and a 50% cut in 2005 carbon emissions by 2050 as primary aims [8]. Consequently, nations were motivated to diversify their energy portfolio to reduce reliance on fossil fuels [9]. However, there are few alternatives to conventional fuels in aviation due to the dependency on high-energy density liquid fuels [10].

In this context, greater efforts should be made in the development and deployment of alternative fuels, gradually replacing Jet A1 [11]. Biomass-derived jet fuel (biojet) has become a key element in the aviation industry’s strategy to reduce environmental impacts. Four major catalytic processes have been investigated for producing biojet fuel: (1) alcohol-to-jet (ATJ, alcohol dehydration and oligomerization), (2) oil-to-jet (OTJ, deoxygenation and hydrocracking of triglycerides), (3) gas-to-jet (GTJ, gasification and Fischer–Tropsch synthesis), and (4) sugar-to-jet (STJ, biological/catalytic upgrading of sugars) [12,13,14]. Among these technologies, the hydroprocessing of esters and fatty acids (HEFA) from non-edible and edible oils makes the oil-to-jet technology prevalent in the production of renewable hydrocarbons in the C_8_-C_16_ range, characteristic of the Jet A1 fuel. However, this technology has a relatively high cost level; thus, further studies in this area are justified [15,16].

To lower the costs in biojet production, scientists are currently moving towards the use of waste triglyceric biomass as one of the potential feedstocks for the alternative and sustainable production of renewable hydrocarbons [17]. In catalytic thermal processes, heterogeneous acid catalysts from transition non-noble and noble metals, such as Ni, Mo, Co, Pt, and Pd, supported or unsupported on alumina oxides, have shown promising results, favoring deoxygenation, isomerization, aromatization, and cracking reactions of waste triglyceric biomass [18,19].

The production of renewable hydrocarbons from triglyceric biomass can take place by means of one single step or by a combination of three different deoxygenation reaction pathways: decarboxylation (DCO_2_), decarbonylation (DCO), and/or hydrodeoxygenation (HDO), but with different selectivity in each reaction route. The DCO_2_ and DCO pathways convert free fatty acids (FFA) to paraffins and olefins of straight chain by releasing CO_2_ and CO without the need for high H_2_ pressures. The paraffins and olefins that are produced by DCO_2_ and DCO contain odd numbers of carbons in their chains. In contrast, the HDO pathway converts FFA to paraffins with even numbers of carbons by converting the FFA over high H_2_ pressure and releasing water [20]. Combustible gases, like H_2_, CH_4_, C_2_H_4_, and C_2_H_6_, are also released in the deoxygenation reactions [21].

In recent studies, Bezergianni et al. investigated the hydroconversion of heavy atmospheric gas oil (HAGO) and waste cooking oil (WCO) mixtures (70:30) over Co-Mo/Al_2_O_3_ and Ni-Mo/Al_2_O_3_. The organic liquid products (OLP) that were obtained from the catalysts achieved around 45% (Ni-Mo/Al_2_O_3_) and 55% (Co-Mo/Al_2_O_3_) conversion into renewable hydrocarbons at 330 °C, 812 psig, liquid hourly space velocity (LHSV) = 1 h^−1^, and H_2_/Oil = 505.9 NL/L [22]. Guatam & Vinu studied, through catalytic fast pyrolysis (CFP), the deoxygenation of *Nannochloropsis oculata* microalgae using Co-Mo that was supported on γ-Al_2_O_3_, and showed 39% selectivity into renewable hydrocarbons in the OLP that was obtained at 500 °C. The pyrolysis experiment was performed with a catalyst-to-algae mass ratio of 1:1 [23]. On the other hand, Souza et al. evaluated the thermal conversion of cottonseed oil dreg into renewable hydrocarbons, and showed that the Mo catalyst that was supported on titanium oxide yielded around 11% of renewable hydrocarbons when the micropyrolysis is performed at 500 °C, while, for the micropyrolysis conducted at 550 °C, the yield was 34% [6]. In addition, Mo, Co, and Ni catalysts have also been used to study the deoxygenation of FFA, such as: Ni-Mo and Co-Mo supported on γ-Al_2_O_3_, used in the deoxygenation of oleic acid [24], unsupported Co-Mo for decarboxylation of the same acid [25], and Mo carbide, which is used in the hydrodeoxygenation of stearic acid [26].

Although conventional chromatography techniques, such as Gas Chromatography/Mass Spectrometry (GC/MS) and Gas Chromatography with Flame Ionization Detector (GC-FID), can be used to identify and quantify the light fraction of bio-oils, they cannot detect the components of high molecular mass that correspond to the heavy fraction of the bio-oil [27,28]. To solve this, analytical techniques based on High-Resolution Mass Spectrometry (HRMS) were developed as complementary tools for bio-oil characterization [29,30]. Fourier Transform-Mass Spectrometry (FT-MS) techniques, combined with Electrospray Ionization (ESI) mode, which use Orbitrap-MS and Ion Cyclotron Resonance (ICR-MS) mass spectrometry analyzers, have successfully been used in the advanced characterization of semivolatile, nonvolatile, and high molecular weight molecules and compounds present in bio-oils that are derived from different biomass feedstocks, such as: spruce wood, poplar wood, beech wood and miscanthus [31], soursop seed cake and bocaiuva seed cake [32], aquatic invasive plants [33], and hydrogenated palm oil [34]. Benés et al. reported that the characterization of bio-oils by ESI-FT-Orbitrap MS can help to establish the main reaction pathways that occur in the hydrodeoxygenation of bio-oils derived from pine wood pyrolysis [35].

The main objective of this work is to produce renewable hydrocarbons with potential application in the aviation sector. In this context, waste cottonseed oil (WCSO) was subjected to non-catalytic and catalytic micropyrolysis experiments at 500 °C. Chemical modifications that were caused in the liquid (bio-oils) and gaseous fractions by downstream upgrading of pyrolysis vapors using Mo-Ni and Mo-Co catalysts supported on γ-Al_2_O_3_ in comparison to the non-catalytic process were evaluated by an in-depth characterization of the liquids employing the following techniques and physicochemical analyses: elemental analysis (CHN), GC/MS, GC-FID, micro-GC with thermal conductivity detector (µGC-TCD), and acid value measurements. In addition, a comprehensive molecular analysis of semivolatile, nonvolatile, and high molecular weight compounds was carried out on the different bio-oils that were derived from WCSO pyrolysis with and without catalytic upgrading, by means of ESI(±)-FT-Orbitrap MS.

The present work innovates in the conversion of a waste triglyceric raw material (WCSO) into renewable hydrocarbons, and the general objective is to obtain preliminary results of a proof-of-concept for continuous catalytic pyrolysis at the microscale that are able to evaluate the potential of catalysts for this purpose. Another highlight of the work is the analysis of bio-oils by ESI(±)-FT-Orbitrap MS, which provides a new way to investigate the influence of catalysts on the catalytic pyrolysis of triacylglycerols. Currently, no studies have yet reported the effect of catalysts over the heavy compounds (polar compounds > 500 Da) present in the liquid fractions from triacylglycerols pyrolysis that can be produced by secondary reactions that present condensate structures of fatty acids. There are limitations in the analysis by conventional techniques of this class of compounds, such as gas chromatography, [36,37], and the use of direct infusion techniques that are associated with mass spectrometry are required to characterize these compounds inside the pyrolysis liquid fraction [33,35].

## 2. Materials and Methods

A sample of cottonseed oil dregs (CSOD) was provided by the experimental biodiesel plant of the Northeast Strategic Technologies Center (CETENE, Caetes, Pernambuco, Brazil). Centrifugation employing a relative centrifugal force (RCF) of 1569·g during 5 min. determined the oil content in the CSOD. The oil obtained was called waste cottonseed oil (WCSO), and it used for the purposes of this work.

### 2.1. Catalysts Preparation

Two supported Mo-Co and Mo-Ni catalysts were prepared by incipient wetness impregnation using commercial high-purity γ-Al_2_O_3_ (Spheralite 505, Axens Procatalyse Catalysts & Adsorbents) as support. The catalysts were prepared while using ammonium heptamolibdate ((NH_4_)_6_Mo_7_O_24_) and nitrate hexahydrate salts of Co (Co(NO_3_)_2_·6H_2_O) and Ni (Ni(NO_3_)_2_·6H_2_O), which were used as Mo, Co, and Ni precursors, all being high-purity (>99.0%) reagents supplied by Sigma–Aldrich. Deionized water (Milli-Q) was used as the impregnation solvent, in which the necessary amounts of the precursor salts were dissolved to obtain nominal metallic contents of 15 wt.% of Mo and 3 wt.% of Co or Ni in the final solids after calcination. As an example, to prepare the 15 wt.%Mo-3 wt.%Co supported on γ-Al_2_O_3_ (Mo-Co/γ-Al_2_O_3_), 12.3 g of ammonium heptamolibdate and 5.0 g of support were used in the impregnation. The volume of deionized water that was needed for preparing the Mo solutions for the impregnations was determined by taking the pore volume of the support measured by the controlled addition of deionized water into a sample until the complete filling of the pores into account. After impregnation with the Mo precursor, solids were dried (105 °C on stove for 12 h) and later calcined at 300 °C for 12 h in a Nabertherm B180 muffle furnace. Subsequently, the Co precursor was incorporated onto the catalyst, again by incipient wetness impregnation, while using Co(NO_3_)_2_.6H_2_O as precursor. In addition, another catalyst was prepared by the sequential impregnation with Mo and Ni also supported on γ-Al_2_O_3_ (Mo-Ni/γ-Al_2_O_3_). After depositing Mo, the solids were dried at 105 °C for 12 h and subsequently calcined at 500 °C for 6 h. The resulting solids were subjected to impregnation of the Ni precursor and, again, underwent the same drying and calcination steps. Finally, all of the catalysts were ground and sieved, selecting the fraction with particle sizes between 100 and 200 µm. The metallic contents of Mo, Co, and Ni in the catalyst samples was determined by Inductively Coupled Plasma-Optical Emission Spectrometry (ICP-OES) by the Servicio de Apoyo a la Investigación (SAI) of the Universidad de Zaragoza (Zaragoza, Spain). The γ-Al_2_O_3_ used as catalyst support and α-Al_2_O_3_ (Strem Chemicals) were both also explored to evaluate their catalytic potential in the upgrading of pyrolysis vapors [38].

### 2.2. Catalyst Characterization

The textural properties of the catalysts and the standalone aluminas were determined by means of adsorption–desorption measurements of N_2_ at 77 K. This technique allowed for determining the parameters of specific surface area (m^2^ g^−1^), specific total pore volume (cm^3^ g^−1^), pore size distribution, and mean pore diameter (nm). The most widely used method for determining the specific surface of porous materials is that put forward Brunauer, Emmett, and Teller (i.e., the BET method) has been employed in this work [39]. The adsorption–desorption isotherms of N_2_ were determined at the normal boiling temperature of liquid nitrogen, −196 °C (77 K), using a static method on a Micromeritics Gemini V automatic volumetric analyzer. Before analysis, the samples were treated at 200 °C for 2 h in N_2_ flow. The surface areas were calculated using the BET equation with five values of N_2_ adsorbed at relative pressures between 0.05 and 0.25.

Temperature-programmed reduction (TPR-H_2_) analyses were carried out in a Micromeritics Autochem 2920 kit. The sample (about 50 mg) was placed, forming a bed of particles in a U-shaped quartz tube that is introduced into an oven. A stream that was composed of 5% H_2_ in Ar was passed through the bed with a flow rate of 75 mL N min^−1^. The sample was heated from room temperature to 950 °C by a ramp at 5 °C min^−1^. The condensable gases produced were retained by means of a liquid N_2_/isopropanol cold trap upstream of the thermal conductivity detector (TCD). The operating parameters were adjusted to meet the criterion that was established by Malet and Caballero [40]. These authors defined a parameter (*P*) given by Equation (1) whose value must be less than 20 for the RTP-H_2_ analysis to develop properly. Where: *β* = heating rate (K min^−1^), *α* = stoichiometric coefficient of H_2_ in the reduction reaction, *S*_0_ = initial amount of reducible species (µmol), *F* = total flow of gas used (cm^3^ min^−1^), and *C*_0_ = H_2_ concentration in gas (µmol cm^−3^).

(1) $$P=\frac{\beta \cdot a\cdot S_{0}}{F\cdot C_{0}}$$

The X-ray diffraction (XRD) measurements were carried out in the X-ray Diffraction and Fluorescence Analysis by the Servicios de Apoyo a la Investigación (SAI) at the University of Zaragoza. The data were collected at room temperature using a RIGAKU brand diffractometer, model D/max 2500, which was working at 40 kV and 80 mA with a copper anode and a graphite monochromator (Cu Kα, λ = 1.5405 Å). The XRD patterns were recorded in a 2θ angle range from 3 to 85° with a scanning rate of 0.03° s^−1^.

The elemental analysis of the catalysts was executed by means of optical emission spectrometry with inductively coupled plasma (ICP-OES). The analyses were performed by the Servicios de Apoyo a la Investigación (SAI) at the University of Zaragoza. The apparatus employed is a Thermo Elemental IRIS INTREPID RADIAL, equipped with a Timberline IIS automatic. The dissolution of the sample has been carried out by means of a microwave-assisted acid digestion.

### 2.3. Characterization of the Waste Cottonseed Oil

The WCSO density was determined to find the mass of biomass that was processed at 500 µL, employing a portable density meter from Kem Kyoto electronics, model DA-130N, at 25.4 °C. The moisture and ash contents were analyzed according to ASTM D1762-84:2007 [41], employing a mass of 1.0000 g of WCSO. The moisture content was calculated from the mass loss after heating at 105 °C for 12 h. The ash content was determined by difference, after subjecting the samples to heating at 750 °C for 6 h. All of the analyses were performed in triplicate.

The thermal behavior of the WCSO was evaluated by thermogravimetric analysis (TG) and differential thermal gravimetric analysis (DTG), employing a Shimadzu TGA-50 analyzer. A 6.9 mg mass of sample was heated at a rate of 8 °C min^−1^ in the range 18–600 °C, under a nitrogen atmosphere at a flow rate of 40 mL min^−1^.

### 2.4. Non-Catalytic and Catalytic Off-Line Micropyrolysis

**Figure 1:**
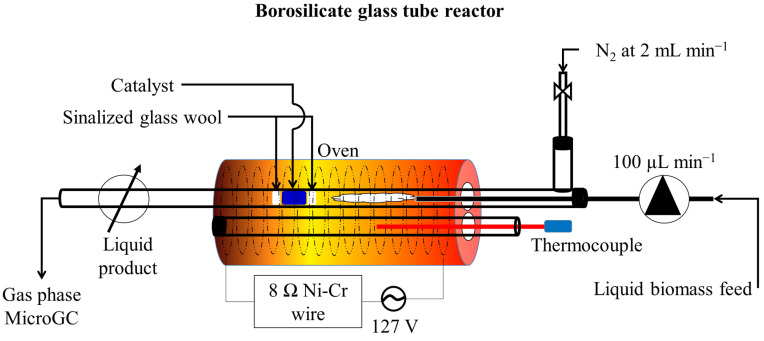
Schematic diagram of the catalytic micropyrolysis system for waste cottonseed oil pyrolysis.

The continuous off-line micropyrolysis *ex-situ* system was constructed in-house, and it is schematically shown in Figure 1. A borosilicate glass tube (450 mm × 5 mm) was used as the pyrolysis reactor. The tube was placed in a clay furnace with 50 mm ID and 300 mm length and then wrapped with nickel-chromium wire, which was equipped with a temperature controller to maintain set-point temperature, and an infusion pump with a feed flowrate control to feed the WCSO liquid. Pyrolysis vapors were condensed through a system that consisted of one cold-trap on the outlet of the pyrolysis reactor at 5 °C.

Prior to each experiment, the reactor was placed in the furnace and then heated up to 500 °C while being purged with pure N_2_ at 200 mL min^−1^ for 5 min. to remove any traces of O_2_ from the system. After the reactor reached 500 °C, the N_2_ purge was set at a constant flow rate of 2 mL min^−1^, and the feed pump was started at a set flow rate at 100 µL min^−1^. The system was operated in continuous mode for 5 min. In that time, 459 mg of WCSO was processed. On each catalytic micropyrolysis experiment, 200 mg of catalyst was used, which was confined within silanized glass wool plugs.

At the end of the experiment, the reactor furnace was turned off and allowed to cool down to room temperature. Thereafter, the liquid products were collected in vials of 2.0 mL and then analyzed by gas chromatography with a flame ionization detector (GC-FID) to check the conversion yield of the WCSO into renewable hydrocarbons. The gaseous product was collected in a 2.5 L Latex gas bag and immediately analyzed using an Agilent 490 Micro-GC Biogas Analyzers system with a thermal conductivity detector (TCD). All of the non-catalytic and catalytic micropyrolysis experiments were performed per triplicate.

### 2.5. Product Gas Characterization

Analysis and quantification of the non-condensable gaseous products that were derived from non-catalytic and catalytic pyrolysis experiments was performed using an Agilent 490 Micro-GC Biogas Analyzers system with a thermal conductivity detector (TCD). The system consists of a dual channel enclosure, employing a CP-Molsieve 5A column on the first channel and a CP-PoraPLOT U on the second channel; both 10 m long.

In the first column hydrogen (H_2_), oxygen (O_2_), nitrogen (N_2_), and carbon monoxide (CO) were quantified using argon as a carrier gas. In the second column, CO_2_ and light hydrocarbons (C_1_~C_3_) were quantified, using helium as carrier gas. A standard gas mixture consisting of H_2_, N_2_, CH_4_, CO, CO_2_, C_2_H_6_, C_2_H_4_, and C_3_H_8_ was used to calibrate the instrument to quantify the yield of non-condensable gases. Gas molecules with more than three carbon atoms (>C_3_) were either not detected or negligible in this research. The operating conditions were as follows: injector temperature of 50 °C, with injection of 20 ms. The initial pressure in the first channel was 25 psi and in the second of 20 psi. The oven temperature was 70 °C and 50 °C in the first and second modules, respectively. The analysis time was 140 s.

### 2.6. Bio-Oil Characterization

#### 2.6.1. Gas Chromatography with Flame Ionization Detector

The analysis and quantification of the bio-oils obtained from the catalyzed and non-catalyzed pyrolysis procedures was performed using gas chromatography, employing a GC-FID instrument Model 7890A, LECO/Agilent. The column used was a ZB-5 (30 m × 0.25 mm ID, 0.25 μm). The operating conditions were, as follows: detector temperature of 320 °C; isothermal injection at 280 °C, inlet operated in split mode (1:40), with an injection of 1.0 μL; hydrogen as carrier gas at a constant flow rate of 1 mL min^−1^; and, oven temperature program of 50 °C for 2 min, followed by heating at 6 °C min^−1^ up to 300 °C (held for 15 min).

The quantification was performed employing a (Jet-A1) commercial kerosene sample as external standard. A stock solution at 75.0 mg mL^−1^ in *n*-hexane was used to prepare five working standards at concentrations of 3.0, 6.0, 9.0, 12.0, and 15.0 mg mL^−1^. Concentrations of bio-oils solutions were prepared at 10.0 mg mL^−1^ in *n*-hexane. An analytical curve was constructed by the total areas of all chromatographic peaks that were detected for the Jeat-A1 versus the concentration of each standard solution, which was used to determine the bio-oil mass of total volatile organics that can be analyzed by GC-FID. For the determination of the renewable hydrocarbons mass, only the areas that were related to the renewable hydrocarbons produced were considered. The areas corresponding to the oxygenated compounds (FFA and others) that were not effectively pyrolyzed were not considered in the calculation.

#### 2.6.2. Gas Chromatography/Mass Spectrometry (GC/MS)

Solutions of a commercial petrodiesel sample and the bio-oils were prepared at 10.0 mg mL^−1^ in *n*-hexane, and analyzed using a Shimadzu GC/MS system, model GC-2010Plus, fitted with a mass spectrometer TQ-8040, inlet AOC5000Plus. The column used was a Rtx-5Sil MS (30 m, 0.25 mm ID, 0.25 μm). The oven temperature was kept at 50 °C for 2 min., followed by a ramp at 6 °C min^−1^ up to 300 °C (held for 15 min). The carrier gas was helium at a constant flow rate of 1.0 mL min^−1^ (53.5 kPa). The injection volume was 1.0 μL, the split ratio was 1:40, the injector temperature was 280 °C, and the interface temperature was 300 °C. The MS operated at electron ionization mode (70 eV) in a full-scan range (40–550 *m/z*). The total analysis time was 58.67 min. The retention index of the components that were present in the bio-oils was calculated based on the retention time of the n-alkanes of the petrodiesel sample. The chemical identities of the components present in the bio-oils were determined using NIST Spectrotech 107, NIST 21, and Wiley 8 libraries, and through the retention index database of the NIST.

#### 2.6.3. Electrospray-Fourier Transform-Mass Spectrometry (ESI-FT Orbitrap MS)

ESI-FT Orbitrap MS analyses are able to provide the exact masses of hundreds or thousands of molecules that can be present in a pyrolysis bio-oil sample. These masses are converted into a molecular formula, such as C_x_H_y_O_w_, and can even be converted to a structural formula using information from the literature and the Double Bound Equivalent (DBE). The bio-oil samples that were obtained from the non-catalytic and catalytic pyrolysis experiments were analyzed by electrospray ionization Fourier transform mass spectrometry in both positive and negative ion modes (ESI(±)-FTMS). These analyses were performed by dissolving the sample in a toluene/methanol mixture (1:2 *v*/*v*) to produce a final solution of 20 ppm. The ESI-FT Orbitrap MS data were collected in an HCD Exactive Plus mass spectrometer (Thermo Scientific, Bremen, Germany) with the following conditions: capillary voltages at +4.2 and −3.8 kV, S-lens RF level at 40, and capillary temperature at 300 °C. Nitrogen was used as a nebulization gas. The MS acquisition was operated in Full Scan MS mode at a resolution of 140,000 FWHM (full width at half maximum) at *m*/*z* 200 along the *m*/*z* range of 150–1000 Da using the Thermo Xcalibur 3.0.63 software from Thermo Fisher Scientific Inc., and a total of 100 µscan were accumulated in each run. The final mass spectra for each sample were obtained from the blank mass spectra subtraction. A molecular formula match was considered when the mass error between the experimental *m*/*z* and theoretical *m*/*z* from the library value was less than 5 ppm.

#### 2.6.4. Acidity Index Determination

The acidity index of the bio-oil and WCSO were determined according to ASTM D664:2015 [42]. A mass of 100.0 mg of sample was solubilized in 5.0 mL of distilled water:isopropyl alcohol:chloroform (0.5:49.5:50.0) solution. The solution was titrated with a standardized alcoholic solution of 0.0082 mol L^−1^ KOH in isopropyl alcohol, while using 1% phenolphthalein as indicator. A blank containing only the solvents was titrated under the same conditions. The acidity index (expressed in mg KOH g^−1^) was calculated using Equation (2), where: *V_blank_* = volume (in mL) of KOH solution used in the blank titration; *V_sample_* = volume (in mL) of KOH solution used in the titration of the sample; and, *N* = normal concentration of the alcoholic solution of KOH. The acidity index determinations were performed in triplicate.

(2) $$\mathrm{Acidity}\;\mathrm{index}=\frac{(V_{sample}-V_{blank})\cdot N\cdot 56.11}{\mathrm{Mass}\;\mathrm{of}\;\mathrm{sample}\;\left(g\right)}$$

#### 2.6.5. Elementary Analysis (CHN)

The elemental analysis of the commercial petrodiesel and kerosene sample, WCSO and bio-oils derived from non-catalytic and catalytic pyrolysis experiments were performed in a LECO CHN628, and the results were treated on CHN628 Software ver. 1.30. The equipment was operated with Helium (99.995%) and Oxygen (99.99%) with the furnace temperature at 950 °C and afterburner temperature at 850 °C. Other parameters were adjusted to better sensibility. The equipment was calibrated with Standard EDTA (41.0% C, 5.5% H and 9.5% N) using the mass range between 10–200 mg. The samples were analyzed using 60.0 mg in a Tin Foil. The analysis of each sample was performed per triplicate.

## 3. Results

### 3.1. Catalysts Characterization

Table 1 summarizes the actual metallic content in the Mo-Co/γ-Al_2_O_3_ and Mo-Ni/γ-Al_2_O_3_ catalysts according to the ICP-OES analyses as well as the BET surface areas of all the solids tested.

It can be observed that, in both cases, the incorporation of Mo onto the supports was not complete, which could be due to the fact that Mo loading was carried out in a single impregnation step, thus not being adequately incorporated onto the porous structure of the support. However, the incorporation of the metals was somewhat worse for the Mo-Ni, with the Mo and Ni contents differing significantly from their respective nominal values.

Nonetheless, the impregnation of the metal precursors followed by calcination resulted in a reduction of the surface area of both catalysts. The Co content in the Mo-Co catalyst was closer to the nominal value of 3%, which indicated that the incorporation of this element onto the previously Mo-impregnated precursor was somewhat better and, in turn, it resulted in a much lower surface area of the Mo-Co catalyst when compared to that of the Mo-Ni after the second calcination. As expected, α-Al_2_O_3_ has very low porosity.

XRD analyses of the catalysts (Figure S1, see Supplementary Materials) revealed that both of the catalyst samples are rather amorphous though diffraction peaks corresponding to alumina could be detected in both samples (diffraction patterns 00-029-0063 and 01-077-0396 of the JCPDS-International Centre for Diffraction Data-2000 database, respectively). In the case of the Mo-Ni/γ-Al_2_O_3_ catalyst, no further crystalline phases could be identified, which may be related to the low loadings of Mo and Ni in the catalyst, possibly resulting in Mo and Ni species being present in the form of particles with very low sizes or having amorphous structures, especially in the case of low Mo loadings [43]. Regarding the Mo-Co/γ-Al_2_O_3_ catalyst, a crystalline phase corresponding to a mixed Cobalt Molybdenum Oxide, CoMoO_4_ (JCPDS diffraction pattern 00-021-0868), could also be identified.

Regarding TPR analyses (Figure S2, see Supplementary Materials), the reduction profiles of the two catalysts presented a similar evolution over temperature. In both cases, two distinct reduction events could be observed by the appearance of two peaks, one at low temperatures and another at much higher temperatures. For the Mo-Co/γ-Al_2_O_3_ catalyst, the low temperature reduction peak was found in the 400–500 °C range (peak maximum at ca. 425 °C), whereas the high temperature peak ranged between 620 and 830 °C approximately (peak maximum at around 760 °C). For Mo-Ni/γ-Al_2_O_3_, the low-temperature peak coincided, but presented a different evolution of the TCD signal before the appearance of this peak, which could reveal the existence of another reduction event in the 250–350 °C interval. In addition, the maximum of the high-temperature peak was found at a much higher value (around 855 °C), with this maximum being somewhat broader in comparison to the homologous of the Mo-Co catalyst. It could be proposed that the low-temperature peak found in both samples at around 400–500 °C would correspond to the reduction of Mo oxides, in the form of octahedral Mo^6+^ oxide [44]. The broad region at temperatures around 300 °C observed in the Mo-Ni catalyst would correspond to the reduction of Ni species with very low degree of interaction with the support, mainly as bulk NiO [45]. Thus, the high temperature peak found in both catalysts would be mainly related to the reduction of Mo^4+^ species into metallic Mo. The broad region between the low and high temperature reduction peaks that were observed in both samples could be attributed to the reduction of different Ni and Co species with varying degrees of interaction with the support. Another possibility is the reduction of mixed oxides containing Mo. Small Ni particles are more difficult to reduce than bulk NiO [45], which could correlate with the results that were found in the XRD and ICP-OES analyses. Regarding Mo-Co, it is known that reduction of Co oxides also takes place in two steps [45,46], which could result in overlap of the reduction events for Mo and Co species and, thus, explain the broad region in the 450–650 °C interval. In addition, mixed Co oxides with other metal oxides are more difficult to reduce, so the high reduction temperature peak could not only be due to the reduction of Mo oxides into metallic Mo, but also to the reduction of the mixed CoMoO_4_ oxide, which would also agree with the results from XRD analysis.

### 3.2. Characterization of the Waste Cottonseed Oil

The oil content that was obtained by centrifugation from CSOD was 17.8 wt.%. Table 2 shows the physicochemical properties of the WCSO. As can be seen in the table, these measurements are in agreement with previous data from the literature on refined cottonseed oil. After separating the WCSO and the dregs, the moisture content (0.47 wt.%) and ash (0.19 wt.%) showed that water and inorganic compounds that were mainly associated with the sodium salts, which formed by the combination of free fatty acid and sodium hydroxide during the pretreatment of the cottonseed oil, are retained in the cotton dregs [6]. Therefore, the chemical composition of the WCSO is mainly associated with mono-, di-, or triacylglycerols (TAGs) and FFA (5.3 mg KOH g^−1^), characteristic of acidity index of waste oils (>3.0 mg KOH g^−1^) [47,48].

**Figure 2:**
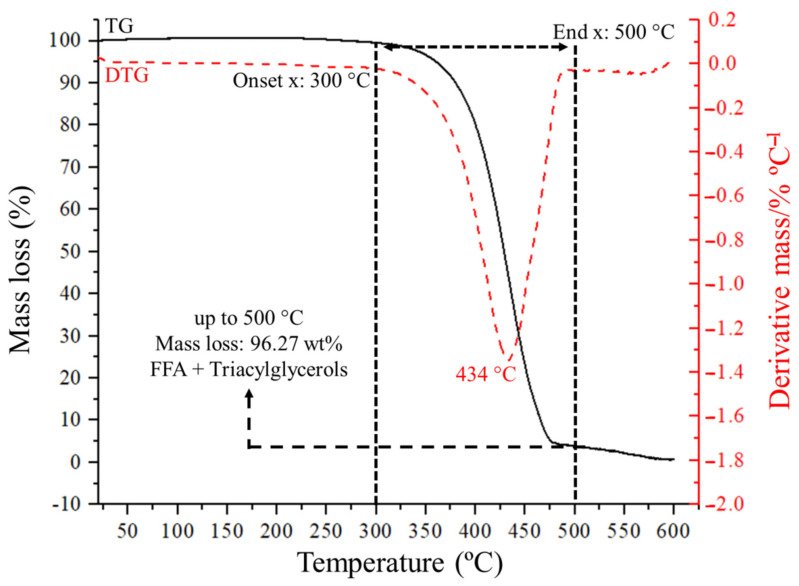
TG curve of the WCSO and its derivative (DTG).

The thermogravimetric analysis (TG) provided information regarding the thermal stability of the WCSO. Figure 2 illustrates the TG curve and its derivative (dotted line), and the thermal behavior of the sample during the heating process. The DTG curve helps to determine the temperature at which there is a maximum rate of sample decomposition in the pyrolysis process, which is located at 434 °C, and that correlates well with the values that correspond to waste cottonseed oil [54].

The WCSO remained stable up to about 300 °C, with a mass loss of 3% below 350 °C, which was attributed to the volatilization of the FFAs, as shown in Figure 2. This higher thermal stability of the oil is related to its structure, typically composed of triacylglycerol’s (having high molecular weights). The TG curve showed that the range of main weight loss was between 350 °C and 500 °C (93.27 wt.%), which is associated to the thermal decomposition of TAGs [55,56]. This indicates that maximum decomposition ends at around 500 °C. According to these data, the micropyrolysis experiments temperature was set at 500 °C. At higher temperatures, the mass residue was 3.73%. The solid residue formed consisted of ash and carbon.

### 3.3. Chemical Characterization of the Bio-Oils

#### 3.3.1. Elemental Analysis (CHN)

Table 3 shows the elementary compositions of the WCSO, petrodiesel, Jet-A1, and bio-oils derived from non-catalytic and catalytic micropyrolysis experiments. The oxygen content was calculated by difference. The elementary composition values of WCSO indicate essentially high contents of carbon (77.69%), hydrogen (11.59%), and 10.64% of oxygen, with very low contents (0.08%) of nitrogen. These results are comparable with the data reported by Daho and co-workers [57] and Xu and co-workers [48] for the refined cottonseed oil and waste cooking oil, with 77.39 and 76.70% of carbon, 11.90 and 10.50% of hydrogen, 11.10 and 11.80% of oxygen, and <0.30 and unspecified% of nitrogen, respectively.

The removal of oxygen is one of the parameters used to verify the catalytic performance in the deoxygenation of triglyceric biomass products [25]. In general, all of the catalytic bio-oils presented lower oxygen content than non-catalytic bio-oil and the raw material. The Mo-Co/γ-Al_2_O_3_ catalyst (BOMoCo) exhibits the highest oxygen removal efficiency (92.78%) from bio-oil among the tested catalysts and solids, as shown in Table 3. Interestingly, the standalone γ-Al_2_O_3_ support (BOγ-Al) presented a very high oxygen removal efficiency (91.75%), showing that the catalyst’s support by itself has activity to produce renewable hydrocarbons from triglyceric biomass pyrolysis, which is in agreement with the evidence found in a previous work on catalytic steam reforming of glycerol [38]. It is also noteworthy that γ-Al_2_O_3_ presented a much higher oxygen removal efficiency than its α-Al_2_O_3_ counterpart, which only had an oxygen removal efficiency of 49.70% (BOα-Al). This can be ascribed to the much better textural properties of γ-Al_2_O_3_ as compared to α-Al_2_O_3_. In any case, both aluminas had much higher removal efficiencies than that attained in the non-catalytic bio-oil (BOWCSO). Interestingly, despite Mo-Ni having a poorer incorporation of metals onto the support, the bio-oil from Mo-Ni/γ-Al_2_O_3_ catalyst (BOMoNi) only had 10.79% less oxygen removal than the Mo-Co/γ-Al_2_O_3_ one, perhaps due to the good catalytic performance of the support itself.

**Figure 3:**
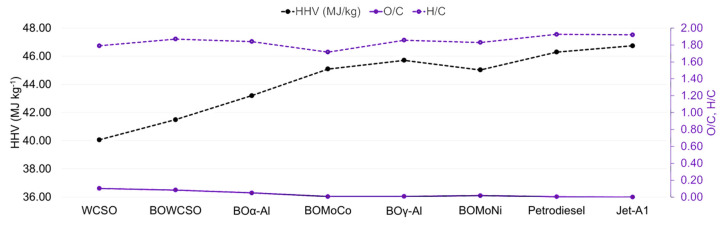
Heating value, O/C and H/C of the feedstock and bio-oils obtained from micropyrolysis experiments at 500 °C.

The heating values of the liquid products were estimated based on the percentage of the elementary composition of the liquid products that are shown in Table 3. As shows Figure 3, WCSO (40.1 MJ kg^−1^) heating value was alike to that of the refined cottonseed oil (39.4 MJ kg^−1^) [52,53]. HHV values for the catalytic and non-catalytic bio-oils were around 41.5–45.5 MJ kg^−1^, while, for petrodiesel and Jet-A1, they are 46.3 and 46.7 MJ kg^−1^, respectively. The HHV values increase for the catalytic bio-oils is attributed to the decrease in oxygen content. Lloyd’s and Davenport’s equation shows that [58]:

HHV (MJ kg^−1^) = 0.3578C + 1.1357H − 0.0845O − 0.0594N − 0.119S

where C, H, O, N, and S are the mass fractions of the corresponding elements. Because the nitrogen and sulfur contents in vegetable oil samples are very low [51], –0.0845(O) is the most significant term contributing to decreased HHV values. Therefore, O removal can generally increase HHV. The bio-oils obtained with the catalysts and with the aluminas decreased the oxygen content and O/C ratio (and C–O, H–O bonds) in the liquid products, thereby increasing the number of C–H bonds (aromatic, saturated, and unsaturated). This results in an increase of the H/C ratio that favors the highest HHV values obtained for BOMoCo, BOγ-Al, and BOMoNi bio-oils (45.09, 45.71, and 45.03 MJ kg^−1^, respectively). Those values are almost the same as petrodiesel and Jet-A1 and higher than the HHV values of alternative fuels, such as the biodiesels that are derived from waste cooking oil, cottonseed oil, and soybean, for which the higher heating values were 39.48, 41.18, and 41.28 MJ kg^−1^, respectively [59].

#### 3.3.2. Acidity Index

**Figure 4:**
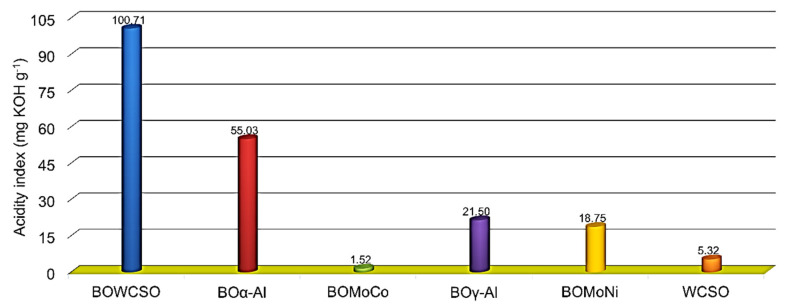
Acidity index of the WCSO and the bio-oils derived from catalytic and non-catalytic micropyrolysis experiments at 500 °C.

Figure 4 shows the acid values of the raw material and the catalytic and non-catalytic bio-oils. An increase on acid values between WCSO (5.32 mg KOH g^−1^) and BOWCSO (100.71 mg KOH g^−1^) of 95.39 mg KOH g^−1^ could be observed. Thus, WCSO’s non-catalytic thermal cracking favors the first step of TAGs degradation, which is associated with the disintegration of the glycerol backbone to produce long chain fatty acids [60]. Other studies on triacylglycerols thermal cracking have reported bio-oils with higher acid values than those of the bio-oils of this work, such as the bio-oil from fish oil (131.1 mg KOH g^−1^) [47], from waste cooking oil (117.8 mg KOH g^−1^ [61] and 126.8 mg KOH g^−1^ [62]), and from cottonseed oil (153.5 mg KOH g^−1^) [54]. All of the catalytic bio-oils showed a decrease in acid values in relation to the non-catalytic bio-oil, which were 18.75 mg KOH g^−1^ for the BOMoNi bio-oil and 21.50 mg KOH g^−1^ for its BOγ-Al counterpart, highlighting the bio-oil obtained with the BOMoCo (1.50 mg KOH g^−1^), while that of BOα-Al bio-oil was 55.03 mg KOH g^−1^. These results are in agreement with the data from the elementary analysis, indicating the high catalytic performance of the Mo-Co/γ-Al_2_O_3_ catalyst in the decarboxylation and/or decarbonylation of the FFAs that formed in the initial step of the thermal cracking processing of the TAGs. This, in turn, is related to the decrease in the O/C ratio, thus raising the higher heating value of the liquid products.

#### 3.3.3. Gas Chromatography/Mass Spectrometry

GC/MS analysis characterized non-catalytic and catalytic bio-oils samples. Bio-oils consist of a complex mixture of different organic compounds derived from the thermal cracking of TAGs and FFAs. The number of peaks detected in the GC/MS chromatograms was 94 compounds for the BOWCSO sample, and 147, 170, 210, and 195 compounds for the BOα-Al, BOMoCo, BOγ-Al, and BOMoNi samples, respectively. The components identified are shown in Table S1 of the Supplementary Materials, and their identification was conducted by comparing the mass spectra of the pyrolysates with the NIST MS and NIST retention index databases. The acidity of the bio-oils that is presented in Figure 4 is attributed predominantly to the presence of the palmitic acid (C_16:0_), linoleic acid (C_18:2_), and stearic acid (C_18:0_) identified, which were also detected in the bio-oils from waste cooking oil [62] and from waste cottonseed oil [54].

**Figure 5:**
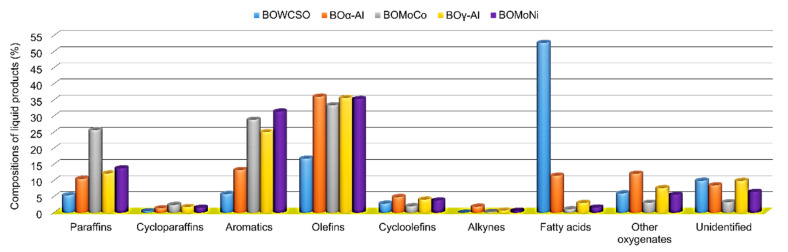
Effect of the different catalysts on the relative compositions of the liquid products.

The bio-oil compositions were grouped in chemical classes from the list of all the compounds that were identified by GC/MS (Table S1, see Supplementary Materials). In general, the relative chemical composition was 31.3, 86.4, and 92.6% of total renewable hydrocarbons and 58.7, 7.2, and 4.2% of oxygenated compounds for non-catalytic bio-oil (BOWCSO), BOMoNi, and BOMoCo, respectively. The renewable hydrocarbons that were derived from BOMoNi and BOMoCo were mainly composed by olefins (35.3 and 33.4%), aromatics (31.4 and 28.9%), and paraffins (13.8 and 25.7%). When the hydrocarbon fraction in the fossil kerosene range is evaluated, relative values of 81.6 and 75.4% (mono-olefins), 45.2 and 43.2% (monosubstituted benzenes), and 27.4 and 50.7% (*n*-pentane) correspond to the olefin, aromatic, and paraffin fractions for the BOMoNi and BOMoCo, respectively. As shown in Figure 5, the relative concentration in paraffins from the organic liquid products followed the trend: BOWCSO (5.4%) < BOα-Al (10.5%) < BOγ-Al (12.2%) < BOMoNi (13.8%) < BOMoCo (25.7%), while the FFA relative concentration decreased from 52.7% without catalyst (BOWCSO) to 1.1% (BOMoCo). This decrease in the decarboxylation of FFA was attributed to the role that is exerted by the catalyst, which decomposes FFA on the surface of catalyst (Mo-Co/γ-Al_2_O_3_), favored by its strong acidity [23,25]. Therefore, catalytic pyrolysis would promote that long chain fatty acids are converted into renewable hydrocarbons. These results agree with the acidity index that is shown in Figure 4 and the oxygen content of elementary analysis presented in Table 3.

Other studies employing Mo, Co, and Ni based catalysts have been reported as promising in the deoxygenation of raw material rich in triacylglycerols. Krobkrong et al. [24] attained deoxygenation of oleic acid employing Co-Mo and Ni-Mo supported on γ-Al_2_O_3_, with yields up to 19 wt.% using both catalysts at the following operating conditions: 330 °C, pressure of 40 bar N_2_ for 3 h. The authors obtained a liquid product with relative chemical compositions of 47/30% and 57/17% at stearic acid and oxygenated intermediates, respectively. However, Moreira et al., in the hydrotreatment from oleic acid and Macauba acid oil, reported 100% and only 34.46% of deoxygenation, respectively, employing a 10 wt.% Co/C, catalyst at 350 °C, and a pressure of 30 bar of H_2_ for 2 h [63].

The BOWCSO showed a chemical composition predominantly consisting of FFAs (52.7%), followed by olefins (16.8%). For the bio-oils that were obtained with the catalysts and their support, the fraction of olefins was the prevalent, with values around 33.4–36.0% (Figure 5). Studies report that the cottonseed oil is mainly composed of unsaturated FFA (73.2%) [64], and, for waste cottonseed oil, this value was 72.2% [6]. Thus, the olefins fraction in catalytic bio-oils mainly comes from the thermal cracking of unsaturated fatty acids. Its mechanism is explained by the disintegration of the glycerol backbone to produce long chain fatty acids, which can be degraded through decarboxylation and/or decarbonylation reactions to produce heavy hydrocarbons (saturated or unsaturated) [60]. Alternatively, the thermal degradation of long chain fatty acids can be attributed to the unsaturated sites that enhance the cleavage of C–C bonds, at a position α, β to the unsaturation, thereby producing short chain fatty acids and light hydrocarbons (saturated or unsaturated). This cleavage mechanism is a dominant reaction [65].

Regarding aromatic composition, this followed the trend: BOWCSO (5.8%), BOα-Al (13.3%) < BOγ-Al (25.1%) < BOMoCo (28.9%) < BOMoNi (31.4%) (Figure 5). These compounds are formed by the Diels–Alder reaction, characterized by the cyclization between a diolefin and an olefin, producing polysubstituted cyclohexenes, which are transformed by dehydrogenation reactions to aromatic compounds [36,60]. Wang et al. reported that the catalytic pyrolysis of fatty acid sodium salts at 500 °C, employing an acid zeolite catalyst (HZSM-5), favors a liquid product with a composition rich in aromatics (33%) [66], which was similar to that of the bio-oils that were obtained with Mo-Co/γ-Al_2_O_3_ (28.9%) and Mo-Ni/γ-Al_2_O_3_ (31.4%) in this work. However, Gautam et al. reported only 6.32% of aromatics in the composition of the liquid product that was obtained from *Nannochloropsis oculata* microalgae via the CFP technique at 500 °C, using a Co-Mo/γ-Al_2_O_3_ catalyst [23].

#### 3.3.4. Gas Chromatography with Flame Ionization Detector

**Figure 6:**
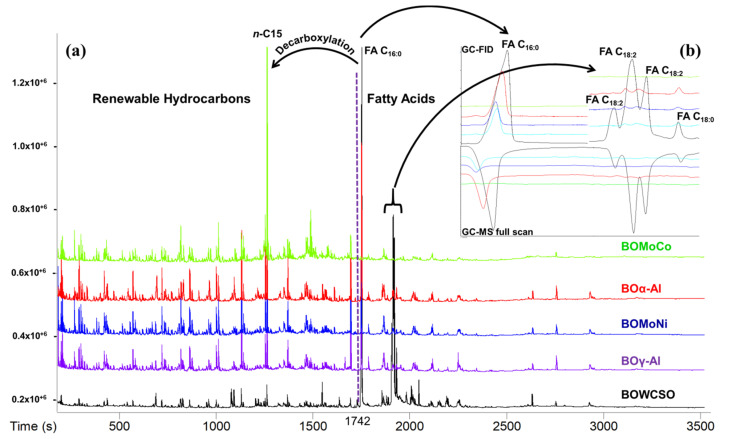
(**a**) Chromatograms of the non-catalytic and catalytic bio-oils obtained by GC-FID and used in the quantification of renewable hydrocarbons and (**b**) FID vs. MS comparison in the range of the retention times for palmitic acid (FA C_16:0_); linoleic acid (and isomers, FA C_18:2_), and stearic acid (FA C_18:0_).

The detection of fatty acids is hindered when analyzed directly, because the hydroxyl of the carbonyl group chemically interacts with the silanols of the stationary phase, which causes the chromatographic band to widen. This leads to difficulties in both the identification and quantification of fatty acids using full scan MS, which has lower sensibility in relation to the FID detector. It must be noted that the fatty acids C_18:2_ and C_18:0_ were only detected in the BOWCSO sample using full scan MS, while, in GC-FID analysis, these fatty acids were detected in the BOWCSO, BOγ-Al, BOMoNi, and BOα-Al samples, as demonstrated in Figure 6b.

Bio-oils, in general, are considered to be a complex mixture composed of hundreds of compounds. Thus, the individual quantification was not feasible. The use of GC/MS is another limiting factor in quantification due to the discriminatory response of the detector for different species of the same class of compounds with different numbers of carbon atoms. The FID detector that is used in chromatography analysis has an advantage due to its response being proportional to the number of carbon atoms and, thus, it can be used with greater accuracy in the quantification of complex mixtures [36]. The conversion yield of the WCSO into renewable hydrocarbons was calculated according to Equation (3), where the mass of renewable hydrocarbons is mass determined by GC-FID, and the initial mass of biomass is referred to the mass of WCSO processed.

(3) $$\mathrm{Yield}\;\mathrm{in}\;\mathrm{renewable}\;\mathrm{hydrocarbons}\;\left(\%\right)=\frac{\mathrm{mass}\;\mathrm{of}\;\mathrm{renewable}\;\mathrm{hydrocarbons}}{\mathrm{Mass}\;\mathrm{of}\;\mathrm{WCSO}\;\mathrm{processed}}\times 100$$

In the catalytic thermal cracking of triglyceric feedstock, the hydrodeoxygenation (HDO), decarboxylation (DCO_2_), and decarbonylation (DCO) reactions can occur simultaneously over FFAs, but with different selectivity towards each reaction route [24]. To estimate the selectivity of the deoxygenation pathways, the C_15_/C_16_ and C_17_/C_18_ ratios has been used [67]. The GC-FID data showed that the highest C_15_/C_16_ and C_17_/C_18_ ratios were obtained for the bio-oils yielded using the bimetallic catalysts, with values of 9.9 and 3.8 for the BOMoNi, and for BOMoCo 18.8 and 7.1, respectively, indicating that the main deoxygenation of WCSO is DCO and/or DCO_2_ reactions on both catalysts. The tested aluminas also showed the main pathway of DCO and/or DCO_2_ reactions, but with lower C_15_/C_16_ and C_17_/C_18_ ratios in comparison to those yielded by the bimetallic catalysts. These data are in agreement with the results that were reported by Krobkrong et al. employing oleic acid as a model compound of lipids and Co-Mo and Ni-Mo catalysts supported on γ-Al_2_O_3_ [24].

**Figure 7:**
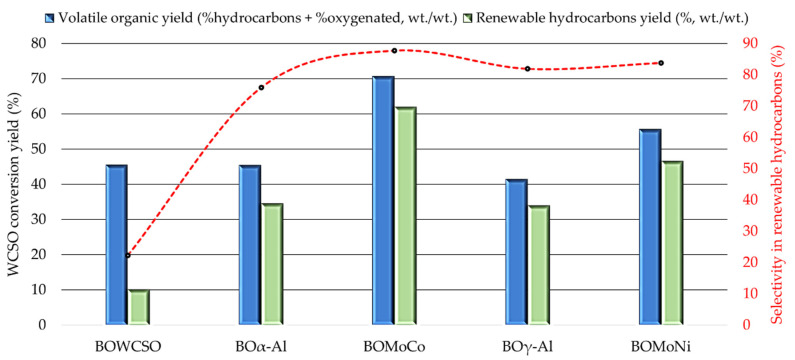
Conversion yields of the WCSO to total volatile organics (%hydrocarbons + %oxygenated) and to renewable hydrocarbons derived from the catalytic and non-catalytic micropyrolysis experiments at 500 °C. Determination performed by GC-FID.

The thermal conversion of the WCSO into renewable hydrocarbons was evaluated using the Mo-Co and Mo-Ni catalysts as well as the standalone aluminas. The yield to renewable hydrocarbons was 10.14% for the non-catalytic process. The deoxygenation reaction over pure α-Al_2_O_3_ and γ-Al_2_O_3_ resulted in similar yields to renewable hydrocarbons, with values around 34.0% (Figure 7). This result, again, confirms that the tested aluminas presented certain catalytic activity for the deoxygenation of WCSO. The wet and dry thermal conversion of cottonseed oil dreg into renewable hydrocarbons has been reported in literature, only reaching a yield of 6% at 500 °C for the dry process, while, for the wet process, the value was 16%. Between the catalytic and non-catalytic wet thermal cracking also performed at 500 °C, there was no increase in the yield to renewable hydrocarbons using a Mo catalyst supported on titanium oxide [6]. When we compared the dry non-catalytic thermal cracking of this literature, our work shows a yield increase of 4.14% in the renewable hydrocarbons production; however, a decrease of 5.86% was observed when compared with the use of a wet biomass. For the wet cracking process, this behavior can be explained by the basic catalysts in the form of salts of fatty acids that are present in the cottonseed oil dregs, which had their catalytic performance enhanced by a greater uniformity of temperature and heat transfer facilitated by the presence of moisture, favoring the thermal cracking of FFA and other chemical species [68]. On the other hand, dry cracking in batch process, in addition to pyrolysis, can promote biomass distillation, which leads to less cracking of the cottonseed oil dreg components. This effect is minimized while employing the continuous pyrolysis process, where biomass is directly fed into the reactor at the programmed pyrolysis temperature (500 °C).

The process yields obtained (Figure 7) clearly showed that the bimetallic catalysts (Mo-Co/γ-Al_2_O_3_ and Mo-Ni/γ-Al_2_O_3_) presented higher conversion yields into renewable hydrocarbons than the standalone aluminas tested, and in relation to the yield obtained in the non-catalytic process. The yield increased from 46.58% (BOMoNi) to 61.94% (BOMoCo). This same behavior was reported in the conversion yield of refined palm olein employing hydrotreatment, increasing from 69.7% (Ni/γ-Al_2_O_3_) to 88.5% (Co/γ-Al_2_O_3_) [69]. Other studies of hydrotreatment at 330 °C of heavy atmospheric gas oil (HAGO) and waste cooking oil (WCO) mixtures (70:30) showed higher conversion yields using Mo-Co/Al_2_O_3_ than Mo-Ni/Al_2_O_3_, increasing from 42% to 56%, respectively [22].

Therefore, the results presented in this work indicate that the increase in the yield to renewable hydrocarbons mainly comes from the contribution of Mo-Co catalyst, which provided higher catalytic activity in the decarboxylation of FFAs than the Mo-Ni catalyst. This has been confirmed by the major peak of *n*-pentadecane (*n*-C_15_) generated by the decarboxylation of the palmitic acid (BOMoCo, see Figure 6). This also indicates the potential of the raw material to produce biokerosene through catalytic pyrolysis processes, because the renewable hydrocarbons (paraffins, cycloparaffins, olefins, cycloolefins, and aromatics) that were identified in the GC/MS analysis were mostly in the C_8_–C_16_ range characteristic of the range of hydrocarbons of the Jet-A1 fuel.

### 3.4. ESI(±)-FT Orbitrap MS Analysis

The ESI(±)-FT Orbitrap MS was used to characterize the WCSO and bio-oils that were derived from non-catalytic and catalytic pyrolysis experiments. TAG and DAG (diacylglycerols) ionized as sodium adducts and the base peaks that were identified in both positive and negative ion modes (ESI(±)-FT Orbitrap MS) for all bio-oil samples. These are shown in Table S2 for negative ion mode and Table S3 for positive ion mode and in Figure S3 (see Tables S1–S3 and Figure S3 that are available in the Supplementary Materials). The ESI(+)-FT Orbitrap MS analysis showed a TAG profile that was derived from a WCSO similar to the cottonseed oil that was characterized by three clusters of C_16:0_C_16:0_C_18:2_ (centered at *m/z* 853.72285), C_16:0_C_18:2_C_18:2_ (*m*/*z* 877.72291), and C_18:2_C_18:2_C_18:2_ (*m*/*z* 901.72260) sodiated molecules, which is: PPL (*m*/*z* 853.72285), PLL (*m*/*z* 877.72291), and LLL (*m*/*z* 901.72260), respectively (P = palmitic acid, L = linoleic acid) (Figure S3, part A, a) [70], while the ESI(−)-FT-Orbitrap MS analysis (Figure S3, part B, a_0_) displays a typical profile of FFA, where the main ions were attributed to the palmitic acid (C_16:0_, *m/z* 255.23250), linoleic acid (C_18:2_, *m/z* 279.23258), oleic acid (C_18:1_, *m*/*z* 281.24822), and stearic acid (C_18:0_, *m*/*z* 283.26386).

The bio-oil that was derived from the non-catalytic thermal cracking (BOWCSO) showed residues of TAG and DAG. Regarding the bio-oils derived from the catalytic processes, the cracking of TAG and DAG was total. The ESI(+)-FT Orbitrap MS profiles of the BOMoCo and BOMoNi pyrolysis liquid products presented similar distributions of detected ions in the mass range of *m*/*z* 160–700, changing the relative abundance of the ions, with the most abundant ions centered in *m*/*z* 160–550 (Figure S3, parts A, d and f). This may indicate that the catalytic thermal cracking of the WCSO leads to similar and more uniformly sized molecules using the Mo-Co/γ-Al_2_O_3_ and Mo-Ni/γ-Al_2_O_3_ catalysts. Note that the ESI(+)-FT Orbitrap MS profiles for these catalysts showed a higher relative abundance of ions that were detected in the mass range of *m/z* 160–300, i.e., compounds of high molecular weights were cracked into compounds of lower molecular weights.

The molecular formulas assigned to the ions highlighted in Figure S3 (part A) were used as a search term for possible structures in the Chemspider or NIST databases, which were associated to saturated oxygenated cyclic compounds, containing five- and six-membered rings. The presence of these saturated rings suggests that, in addition to the intermolecular cyclization mechanism via the Diels–Alder reaction, intramolecular cyclizations from alkyl and alkenyl radicals were also favored as a result of WCSO cracking [71]. It can also be observed in Figure S3 (part A, b, d, and f) the change in structures attributed to the ions corresponding to the peak of higher abundance, and note that this change has led to catalytic bio-oils (e.g., BOMoCo and BOMoNi) with a lower degree of oxygen, showing that the catalysts acted, for example, via dehydration and cracking reactions.

The ESI(−)-FT Orbitrap MS profiles display the composition of all acid chemical compounds present in bio-oils that were obtained from triglyceric raw material that can be deprotonated. Because of the molecular weight of these molecules and the polarity associated with the hydroxyl of the carboxyl group, e.g., some molecules could not be analyzed by GC. As shown in Figure S3 (part B, b_0_), *m*/*z* signals equal to 511.47332, 535.47352, 559.47336, and 561.48916 correspond to high molecular weight acids that cannot be analyzed by GC and are generated in the pyrolysis process, since they were not detected in the WCSO. The last two ions mentioned were generated by the condensation of unsaturated FFAs via the Diels–Alder reaction [37]. The *m/z* value of 559.47336 can be attributed to the reaction between two molecules of linoleic acid, and the *m/z* value of 561.48916 to the reaction between one molecule of oleic acid and another of linoleic acid.

With respect to the class distributions for all of the molecular formulas attributed to the ions after data processing, the main species detected by ESI(−) were O2 and O4 in the non-catalytic bio-oil (BOWCSO). These species are associated with FFAs (O2) and high molecular weight acids (O4). A significant and similar consumption of the classes O2 and O4 was observed in the BOα-Al and BOγ-Al bio-oils. Higher consumption of these classes was observed for BOMoCo and BOMoNi, where the Mo-Co/γ-Al_2_O_3_ catalyst showed a greater catalytic performance in the deoxygenation of the FFAs and dimers of high molecular weight acids. These data agree with the acidity index shown in Figure 4.

The O2-O3-O4 species classes were detected as the main ones in the non-catalytic bio-oil (BOWCSO) that was analyzed by ESI(+), followed by the O1-O5-O6 species that showed significant abundance and a residue of O7 species. After WCSO catalytic cracking, O1 and O2 species were detected as the prevalent in all catalytic bio-oils, followed by O3 species. For the bio-oils BOMoCo and BOMoNi, only residues of the classes of species O4-O5 were detected and a total consumption of the O6-O7 species was noted. On the basis of these results, it is assumed that the tested catalysts converted O2-O3 species into O1 species associated to aldehydes, ketones, or ethers; and the conversion of the O4-O5-O6-O7 species into less oxygenated species with different functional groups, such as O2-O3 species.

**Figure 8:**
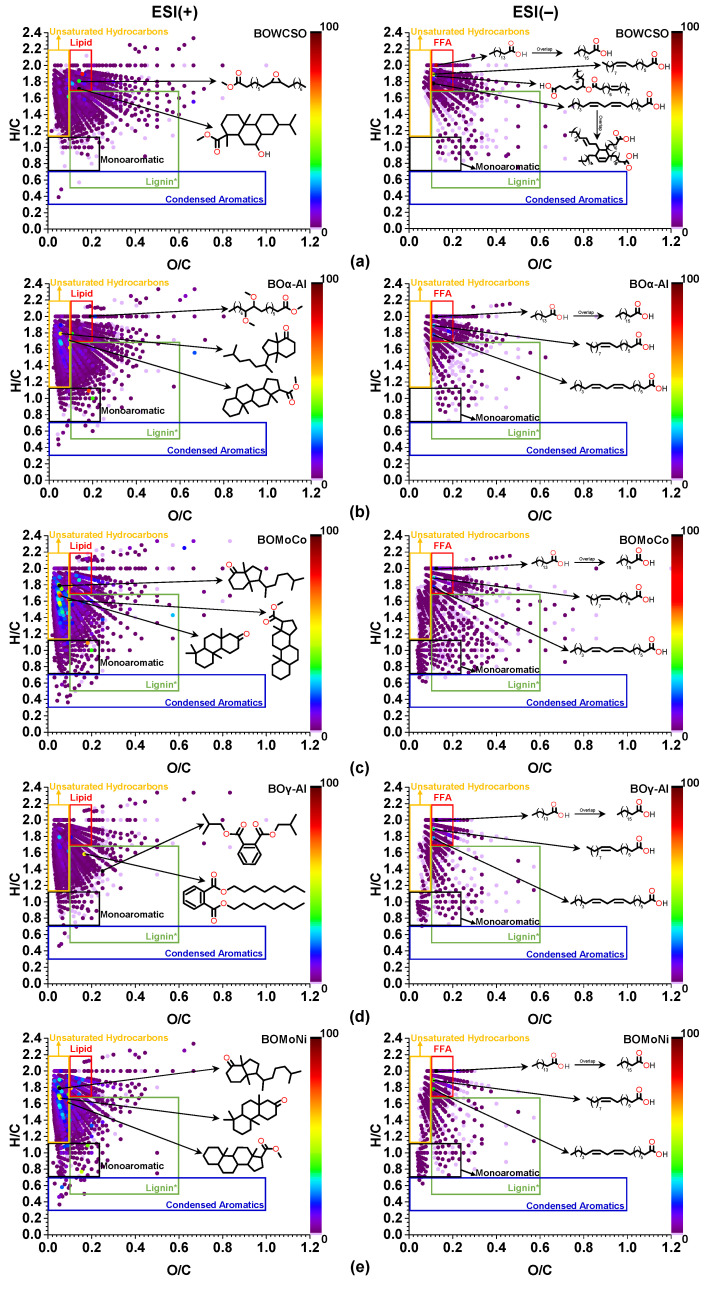
The van Krevelen plot for all bio-oils with C_x_H_y_O_z_ molecules, with (**a**) z = 1–7 in the ESI(+) and z = 2–6 in the ESI(−), (**b**) z = 1–6 in the ESI(+) and z = 2–6 in the ESI(−), (**c**) z = 1–5 in the ESI(+) and z = 1–6 in the ESI(−), (**d**) z = 1–5 in the ESI(+) and z = 1–4 in the ESI(−), (**e**) z = 1–5 in the ESI(+) and z = 1–4 in the ESI(−). Colors purple to black indicate the intensity from 0 to 100.

The representation of the ESI-FT Orbitrap MS results on a van Kravelen diagram is one way of categorizing the components into specific chemical classes in a van Krevelen space [72], in addition to identifying the possible reaction pathways [73]. The van Krevelen space of this work that is shown in Figure 8 was divided into five discrete regions, being modified from the diagrams proposed by Kujawinski & Behn [74], Ohno et al. [75], and He et al. [76]: lipids (H/C = 1.70–2.20 and O/C = 0.10–0.20); monoaromatic (H/C = 0.70–1.10 and O/C = 0–0.22); unsaturated hydrocarbons (H/C = 1.10–2.2 and O/C = 0–0.10); condensed aromatic structures (H/C = 0.30–0.70 and O/C = 0–1.00); and pyrolytic lignin for comparative purposes (H/C = 0.50–1.70 and O/C = 0.10–0.60).

As shown in Figure 8, in ESI(+) the major components in catalytic and non-catalytic bio-oils are of different chemical classes and it was observed that: first, the chemical composition of the non-catalytic bio-oil was mainly attributed to lipids and fatty acid esters (part BOWCSO), which have similar structural characteristics (H/C 1.70–2.20 and O/C 0.10–0.20); second, the cracking of these compounds using the catalysts’ supports led to significant changes in the van Krevelen space, increasing the relative abundance of compounds in the range of unsaturated hydrocarbons, in the range of monoaromatic compounds and in the range of compounds with structural characteristics that are similar to the pyrolytic lignin compounds (part BOα-Al and BOγ-Al); third, the lowest O/C ratio values were obtained for BOMoCo and BOMoNi, being located in the range of unsaturated hydrocarbons (H/C = 1.10–2.2 and O/C = 0–0.10) mainly associated to oxygenated functional groups of one oxygen atom, followed by an increase in relative abundance in the range of monoaromatic compounds (H/C = 0.70–1.10 and O/C = 0–0.22) (part BOMoCo and BOMoNi). This indicates that the tested catalysts favor the reduction of oxygen atoms mainly in the form of CO_2_ (decarboxylation) and CO (decarbonylation) or via reduction reactions; these were probably the main reaction pathways during the catalytic pyrolysis that were identified by ESI(+)-FT Orbitrap MS.

The ESI(–)-FT-ICR-MS analysis has demonstrated that hydrogenation, dehydrogenation, and polymerization reactions are possible reaction pathways in the continuous pyrolysis of plant oil asphalt at 600 °C [72] and in the pyrolysis of plant acidified oil at 500 °C [77]. However, no studies have been reported yet on the analysis of bio-oils derived from the catalytic pyrolysis of TAGs by HRMS of the type ESI(±)-FT-ICR-MS or ESI(±)-FT Orbitrap MS. The van Krevelen plots for ESI(−) that are shown in Figure 8 indicate that, among the aluminas and catalysts tested, the Mo-Co/γ-Al_2_O_3_ catalyst increased the number of monoaromatic compounds (H/C = 0.70–1.10 and O/C 0–0.22) and compounds with structural characteristics that are similar to pyrolytic lignin compounds (H/C = 0.50–1.70 and O/C = 0.10–0.60), which are associated to the presence of aromatic rings in their structures. By evaluating the data from the CHN, GC/MS, GC-FID, and ESI(±)-FT Orbitrap MS analyses, it can be concluded that the Mo-Co/γ-Al_2_O_3_ catalyst showed greater catalytic performance in cracking WCSO. The main conversion routes are decarboxylation reactions, radical cyclization, dehydrogenation, and the reduction of oxygenated functional groups, resulting in a bio-oil having greater quality in relation to the other bio-oils that were derived from the catalytic and non-catalytic pyrolysis experiments.

### 3.5. Chemical Characterization of Pyrolysis Gases

**Figure 9:**
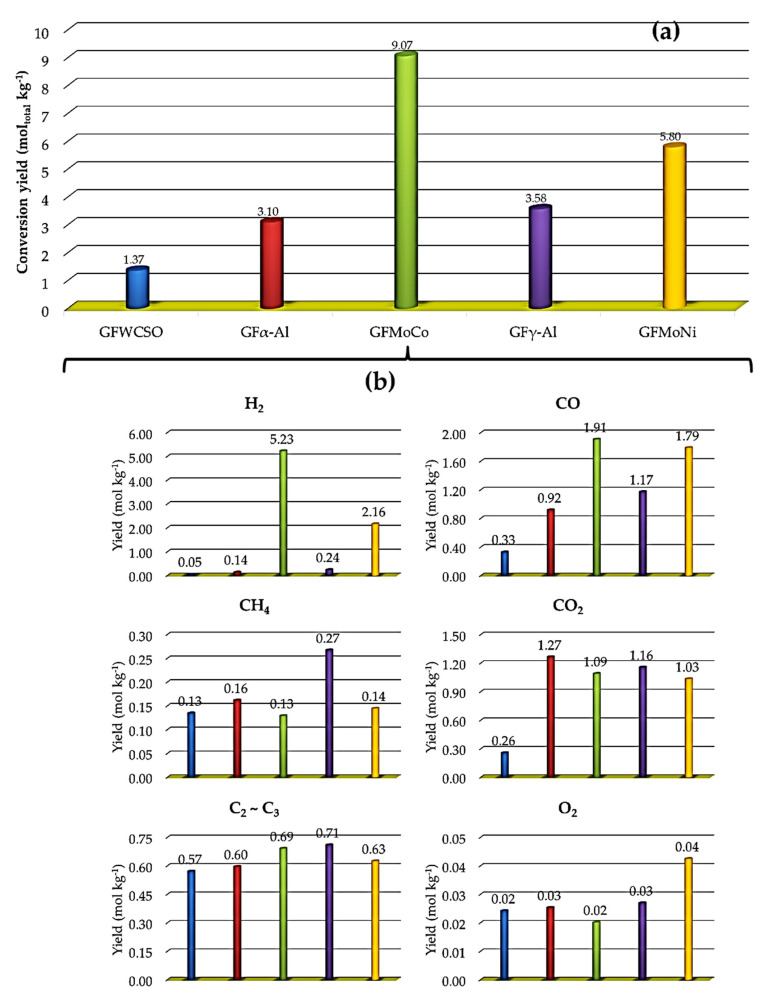
(**a**) Absolute matter amount of gas fractions produced in each micropyrolysis experiment; (**b**) Absolute matter amount of the components that represent the product of gas fraction. Gas compositions are given on a N_2_-free basis.

In Figure 9 (part a), the absolute conversion yields in terms of matter amount per kg of pyrolyzed biomass (mol kg^−1^) of gas fractions (GF) produced is shown. All of the catalytic processes increased the production of pyrolysis gases, in factors ranging from 2.3 (GFα-Al) to 6.6 (GFMoCo) in relation to the non-catalytic process. The highest yields to gaseous products were obtained with the Mo-Ni/γ-Al_2_O_3_ catalyst (5.80 mol kg^−1^), followed by the Mo-Co/γ-Al_2_O_3_ catalyst (9.07 mol kg^−1^).

The quantification of the chemical composition of gaseous products was performed to investigate the catalytic performances of the Mo-Ni/γ-Al_2_O_3_ and Mo-Co/γ-Al_2_O_3_ catalysts and the aluminas that were tested in the catalytic thermal cracking of the WCSO; the result is shown in Figure 9 (part b). The matter amount obtained showed that standalone aluminas had little influence on the generation of H_2_. Conversely, bimetallic catalysts significantly favored the increase of H_2_ produced in relation to the non-catalytic process 1:3:5:43:105 (GFWCSO:GFα-Al:GFγ-Al:GFMoNi:GFMoCo). The dehydrogenation of saturated compounds is the key pathway for H_2_ generation [71], leading to the formation of aromatic compounds. These results are in agreement with the data obtained by GC/MS, with higher production of aromatic compounds on the BOMoCo (28.9%) and BOMoNi (31.4%) samples, but this does not justify the higher relative concentration of aromatics for the BOMoNi, since the highest volume of H_2_ was obtained with the Mo-Co/γ-Al_2_O_3_ catalyst. However, among the tested catalysts and supports, the ESI(+)-FT Orbitrap MS analysis of the BOMoCo showed regions in the van Krevelen space with a greater relative abundance of monoaromatic compounds (H/C = 0.70–1.10 and O/C = 0–0.22) and dehydrogenation of saturated compounds (H/C = 1.10–1.60 and O/C = 0–0.10), which correspond to processes that involve the release of H_2_; and, in ESI(–), an increase in the number of compounds that have similar structures to pyrolytic lignin (H/C = 0.50–1.70 and O/C = 0.10–0.60), which are associated with at least one aromatic ring (Figure 8, part c). Thus, the GC-MS data that are associated with the ESI(±)-FT Orbitrap MS data corroborate the results that were obtained for the volume of H_2_ produced using the Mo-Co/γ-Al_2_O_3_ catalyst.

**Figure 10:**
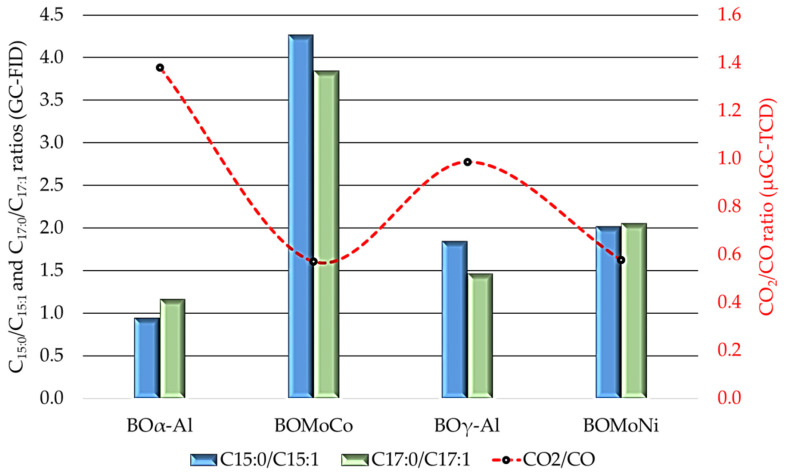
Comparison of the selectivity toward relevant products displayed by the catalysts and supports tested in the decarboxylation/decarbonylation of FFA, by means of representative product ratios (C_15:0_/C_15:1_, C_17:0_/C_17:1_ and CO_2_/CO).

The CO_2_ and CO yields are other parameters that are used to monitor decarboxylation and decarbonylation reactions of FFAs, leading to the formation paraffins and olefins, respectively [78]. In the present study, the CO and CO_2_ matter amount varied, as follows: GFWCSO (0.33 mol kg^−1^) < GFα-Al (0.92 mol kg^−1^) < GFγ-Al (1.17 mol kg^−1^) < GFMoNi (1.79 mol kg^−1^) < GFMoCo (1.91 mol kg^−1^) for the CO; and GFWCSO (0.26 mol kg^−1^) < GFMoNi (1.03 mol kg^−1^) < GFMoCo (1.09 mol kg^−1^) < GFγ-Al (1.16 mol kg^−1^) < GFα-Al (1.27 mol kg^−1^) for the CO_2_. These values indicate CO_2_/CO ratios that were around 0.6 for the two bimetallic catalysts, thus resulting in lower DCO_2_ selectivity and higher DCO selectivity over FFA. In contrast, α-Al_2_O_3_ showed a higher CO_2_/CO ratio (1.4), indicating a higher DCO_2_ selectivity, and γ-Al_2_O_3_ did not have any significant effect on DCO and DCO_2_ selectivity, with a CO_2_/CO ratio that was around 1.0. The selectivity to decarbonylation of the bimetallic catalysts, related to the CO_2_/CO ratio (obtained by µGC-TCD), does not correlate well with the selectivity that was found by the C_15:0_/C_15:1_ and C_17:0_/C_17:1_ ratios (obtained by GC-FID), which mainly indicated FFA decarboxylation reactions (Figure 10, C_15:0_/C_15:1_ and C_17:0_/C_17:1_ > 1). This is justified by the higher H_2_ and CO matter amount and the lower CH_4_ and CO_2_ matter amount produced when using the two bimetallic catalysts in relation to the standalone aluminas tested, which suggests that secondary reactions, such as steam reforming (SR) and the reverse Water-Gas Shift (rWGS) equilibrium, could be taking place [38].

## 4. Conclusions

This work shows the potential of waste cottonseed oil (WCSO) as an alternative source for triacylglycerols that can be valorized via biokerosene production while using catalytic micropyrolysis. Preliminary results on the application and performance of Mo-Ni and Mo-Co catalysts supported on Al_2_O_3_ for upgrading of the pyrolysis vapors were presented. These catalysts promote the WCSO cracking process through DCO and DCO_2_ reactions of FFAs, and by the dehydrogenation of saturated compounds, resulting in a conversion of 46.58% and 61.94% of triacylglycerols waste into renewable hydrocarbons, respectively. A high oxygen removal efficiency could be attained in the upgrading of the pyrolysis vapors with standalone γ-Al_2_O_3_ (91.8%) and with Mo-Co/γ-Al_2_O_3_ (92.8%). Renewable hydrocarbons that were derived from the upgraded bio-oil using the Mo-Co catalyst were mainly composed by olefins (33.4%), aromatics (28.9%), and paraffins (25.7%). Values of C_15_/C_16_ and C_17_/C_18_ paraffins ratios higher than 1 show that the metals present in the catalysts have an important role on decarboxylation reactions, decreasing the presence of residual carboxylic acids and contributing a less acidic pH of the final liquid product. These metals promote aromatization reactions thus improving and increasing the production of aromatic hydrocarbons. Mo and Co especially provide the higher gas yields with quality syngas (H_2_/CO) characteristics. Therefore, the catalytic pyrolysis can be a promising pathway for industrial production of renewable hydrocarbons that were derived from low-cost triglyceric biomass, such as the WCSO, without the need for large amounts of hydrogen supply to conduct the catalytic upgrading treatment.

The reaction pathways that led to the high H_2_ yields that were obtained with Mo-Co/γ-Al_2_O_3_ could only be understood through the joint analysis of the data obtained by GC/MS, ESI(±)-FT Orbitrap MS, and µGC-TCD. The main reactions are aromatization (by GC/MS and ESI(±)-FT Orbitrap MS) and possibly thermal cracking of the WCSO, in combination with steam reforming and reverse Water-Gas Shift as secondary reactions.

Lastly, these results confirm that the use of inexpensive bulk aluminas as potential materials for the upgrading of pyrolysis vapors should be further explored in combination with other catalysts by proposing novel reactor configurations, namely dual bed reactors, among others.

## Figures and Tables

**Table 1:** Metallic content of the catalysts, determined by ICP-OES analysis and surfaces areas of the materials (BET method).

Sample	BET Surface Area (m^2^ g^−1^)	% Mo (wt.%)	% Co (wt.%)	% Ni (wt.%)
Mo-Co/γ-Al_2_O_3_	127 ± 6	8.9 ± 0.4	1.8 ± 0.1	-
Mo-Ni/γ-Al_2_O_3_	234 ± 7	5.0 ± 0.2	-	0.3 ± 0.1
γ-Al_2_O_3_	251 ± 4	-	-	-
α-Al_2_O_3_	3 ± 1	-	-	-

**Table 2:** Physicochemical properties of the waste cottonseed oil.

Property	Reported Value	Reference	This Work
Density (kg m^−3^) ^a^	911.5	[49]	917.2
Moisture (wt.%) ^a^	0.02	[50]	0.47
Ash (wt.%) ^a^	0.0	[51]	0.19
Higher heating value (HHV, MJ kg^−1^) ^a^	39.4	[52,53]	40.1
Acidity index (mg KOH g^−1^) ^b^	10.6	[6]	5.3

^a^ Refined cottonseed oil. ^b^ Oil extracted from the cottonseed oil dregs by liquid-liquid extraction.

**Table 3:** Elementary composition of raw material, petrodiesel, Jet-A1, and bio-oils that were obtained from catalytic and non-catalytic micropyrolysis experiments.

Samples		Elemental Composition (wt.%) *				Oxygen Removal Efficiency (%)
		C	H	N	O ^a^	
Raw material	WCSO	77.69 ± 0.13	11.59 ± 0.30	0.08 ± 0.01	10.64 ± 0.43	–
Non catalytic bio-oil	BOWCSO	78.97 ± 0.15	12.31 ± 0.09	0.00	8.72 ± 0.12	18.0
Catalytic bio-oils	BOα-Al	82.05 ± 0.50	12.59 ± 0.13	0.01 ± 0.01	5.35 ± 0.63	49.7
	BOMoCo	86.81 ± 0.42	12.41 ± 0.05	0.01 ± 0.01	0.77 ± 0.43	92.8
	BOγ-Al	85.81 ± 0.63	13.28 ± 0.11	0.03 ± 0.03	0.88 ± 0.71	91.8
	BOMoNi	85.11 ± 0.54	12.98 ± 0.06	0.00	1.92 ± 0.60	82.0
Reference material	Petrodiesel	85.72 ± 0.17	13.76 ± 0.23	0.00	0.53 ± 0.06	–
	Jet-A1	86.64 ± 0.12	13.86 ± 0.35	0.00	0.00	–

* *n* = 3, values correspond to mean ± standard deviation. ^a^ calculated by difference, disregarding ash values.

## Supplementary Materials

The following are available online at https://www.mdpi.com/article/10.3390/nano11071659/s1, Figure S1. XRD analyses of the Mo-Co and Mo-Ni catalysts. Figure S2. TPR profiles of the Mo-Co and Mo-Ni catalysts. Figure S3. ESI(±)-FT-Orbitrap MS for the raw material and for all bio-oils, (A) positive ion mode and (B) negative ion mode; a, a0 = WCSO (raw material); b, b0 = BOWCSO; c, c0 = BOα-Al; d, d0 = BOMoCo; e, e0 = BOγ-Al; f, f0 = BOMoNi. Table S1. Selectivity of compounds derived from the non-catalytic and catalytic micropyrolysis experiments of WCSO. 1A = BOWCSO, 2A = BOα-Al, 3A = BOMoCo, 4A = BOγ-Al and 5A = BOMoNi. Table S2. Absolute area of some ions detected via ESI(-)-FTMS. 1A = WCSO, 2A = BOWCSO, 3A = BOα-Al, 4A = BOMoCo, 5A = BOγ-Al and 6A = BOMoNi. Table S3. Absolute area of some ions detected via ESI(+)-FTMS. 1A = WCSO, 2A = BOWCSO, 3A = BOα-Al, 4A = BOMoCo, 5A = BOγ-Al and 6A = BOMoNi.
