# Supplementary material for: Renewable Hydrocarbon Production from Waste Cottonseed Oil Pyrolysis and Catalytic Upgrading of Vapors with Mo-Co and Mo-Ni Catalysts Supported on γ-Al2O3
Source: Nanomaterials (Basel). 2021 Jun 24;11(7):1659. doi: 10.3390/nano11071659 (PMC8306218; doi:10.3390/nano11071659)
Supplement: Supplementary file 1 [file nanomaterials-11-01659-s001.zip › nanomaterials-1238776-supplementary.pdf]

Supplementary Material

Renewable Hydrocarbon Production from Waste Cottonseed Oil Pyrolysis and Catalytic Upgrading of Vapors with Mo-Co and Mo-Ni Catalysts Supported on γ-Al_2O_3

Josué Alves Melo [1], Mirele Santana de Sá [1], Ainara Moral [2], Fernando Bimbela [2], Luis M. Gandía [2] and Alberto Wisniewski, Jr. [1,*]

[1] Petroleum and Energy from Biomass Research Group (PEB), Federal University of Sergipe, UFS, São Cristóvão/SE 49100 000, Brazil; josuecedro@hotmail.com (J.A.M.); mirelesantana@live.com (M.S.S.)

[2] Grupo de Reactores Químicos y Procesos para la Valorización de Recursos Renovables, Institute for Advanced Materials and Mathematics (INAMAT[2]), Universidad Pública de Navarra (UPNA), Campus de Arrosadía, 31006 Pamplona, Spain; moral.ainara@gmail.com (A.M.); fernando.bimbela@unavarra.es (F.B.); lgandia@unavarra.es (L.M.G.)

* Correspondence: albertowj@ufs.br

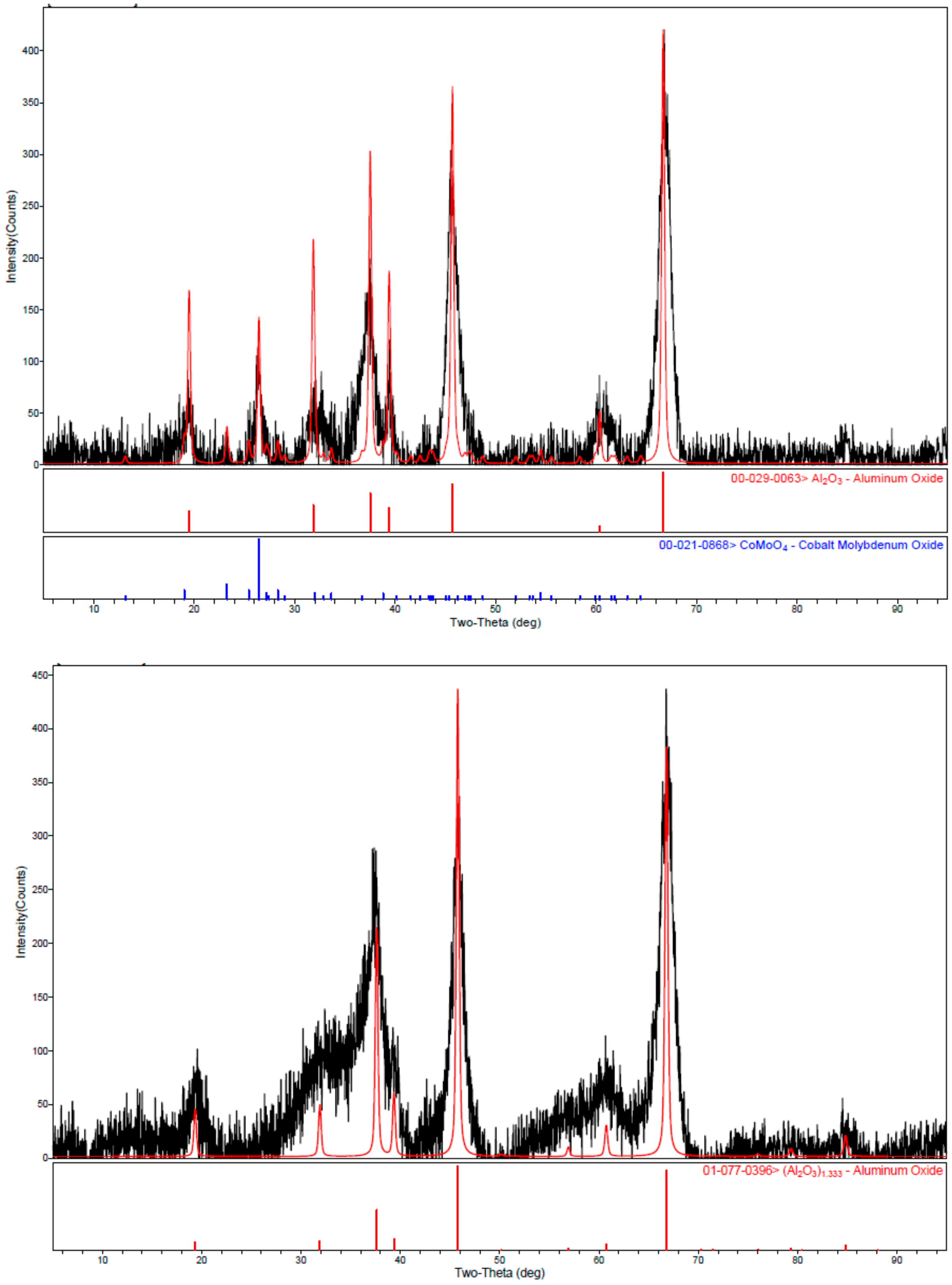

Figure S1. XRD analyses of the Mo-Co and Mo-Ni catalysts**.**

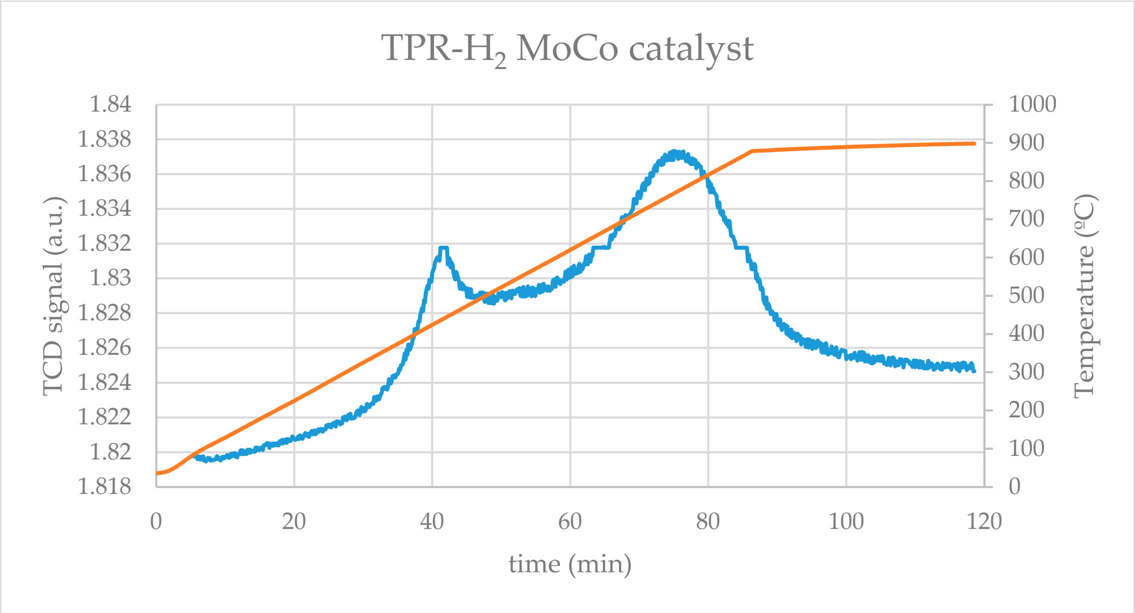

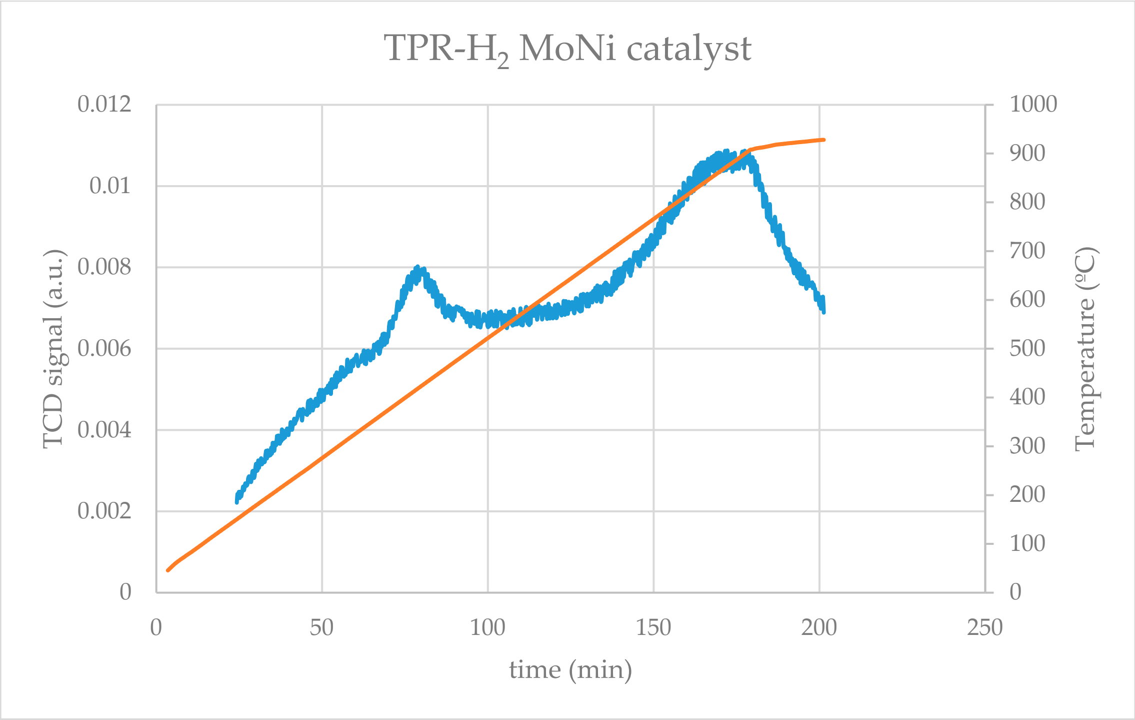

Figure S2. TPR profiles of the Mo-Co and Mo-Ni catalysts.

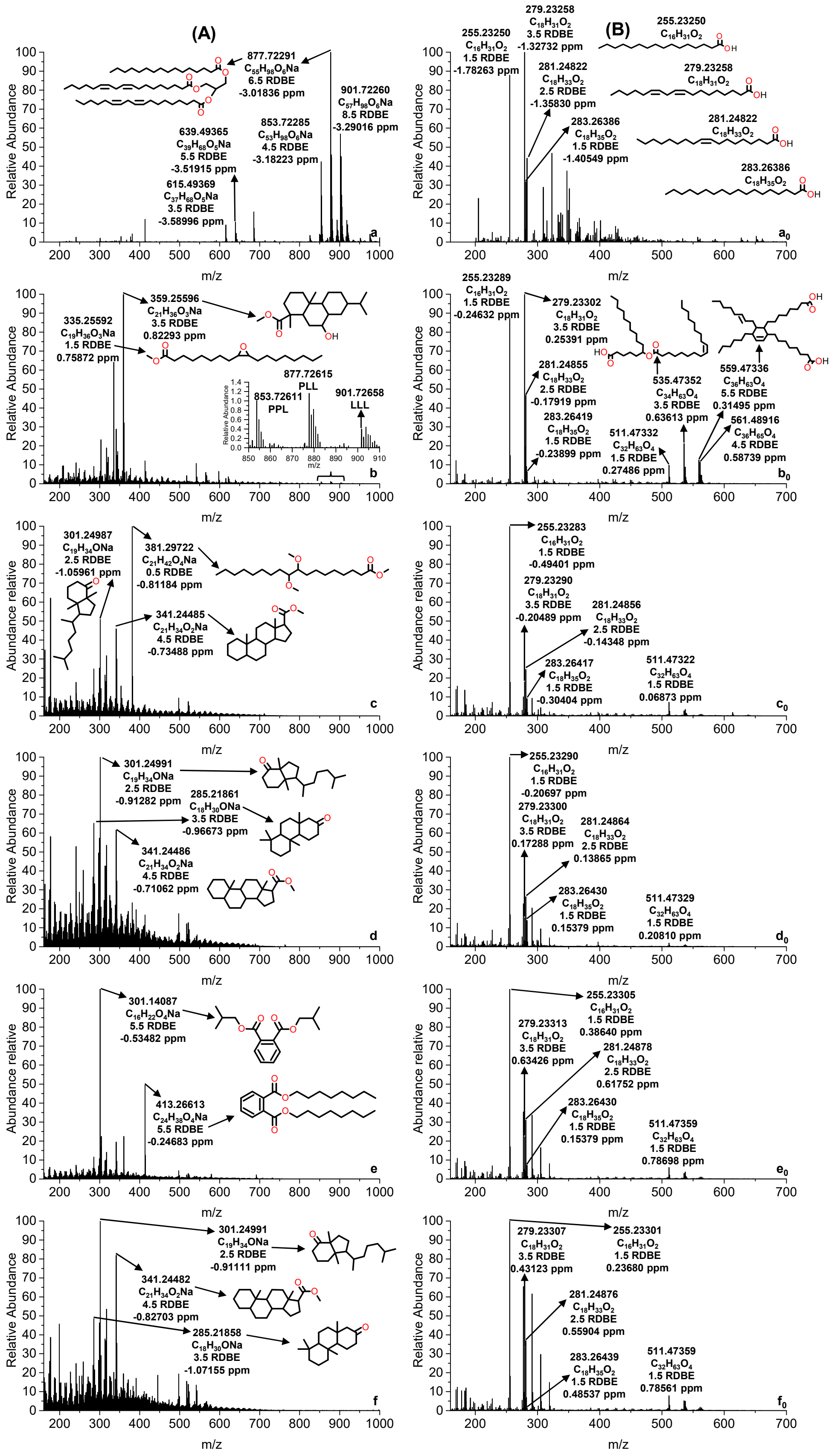

Figure S3. ESI(±)-FT-Orbitrap MS for the raw material and for all bio-oils, **(A)** positive ion mode and **(B)** negative ion mode; **a, a$_0$** = WCSO (raw material); **b, b$_0$** = BOWCSO; **c, c$_0$** = BOα-Al; **d, d$_0$** = BOMoCo; **e, e$_0$** = BOγ-Al; **f, f$_0$** = BOMoNi.

Table S1. Selectivity of compounds derived from the non-catalytic and catalytic micropyrolysis experiments of WCSO. **1A** = BOWCSO, **2A** = BOα-Al, **3A** = BOMoCo, **4A** = BOγ-Al and **5A** = BOMoNi.

Retention Time (min)	Retention Index (NIST)	Compound Name	Relative Area (%)					Retention Index				
			1A	2A	3A	4A	5A	1A	2A	3A	4A	5A
3.127	nf	2-methyl-1,3-cyclohexadiene	–	0.16	–	0.08	0.10	–	774	–	774	773
3.193	775.0	Methylbenzene	0.33	0.85	1.97	1.31	2.93	777	777	777	777	777
3.240	766.4	1-Methylcyclohexene	0.21	0.34	0.27	0.38	0.38	779	779	779	779	779
3.327	780.0	Butanoic acid	–	0.35	–	0.09	0.12	–	784	–	783	783
3.387	nf	1,3-dimethylidenecyclopentane	0.37	0.49	0.09	0.25	0.27	787	787	786	787	787
3.480	793.0	2-Ethyl-1-hexene	0.27	–	–	–	–	791	–	–	–	–
3.520	792.9	1-Octene	1.21	2.21	1.17	1.65	1.84	793	793	793	793	793
3.600	799.0	3-Octene, (Z)	–	–	0.27	0.19	0.32	–	–	797	797	798
3.660	800.0	Octane	0.59	1.05	1.88	1.71	1.89	800	801	800	800	800
3.767	799.0	trans-2-Octene	0.71	1.04	0.88	0.89	1.00	805	805	805	805	805
3.807	812.0	1-Octyne	–	–	0.12	0.17	0.19	–	–	807	808	808
3.907	812.0	cis-2-Octene	0.39	0.46	0.57	0.51	0.60	812	812	812	812	812
4.000	nf	3-Ethyl-1,4-hexadiene	–	–	–	0.09	0.11	–	–	–	817	817
4.127	826.0	1,3-Octadiene	0.70	1.07	0.30	0.63	0.60	823	824	823	823	823
4.280	nf	1-Ethyl-5-methylcyclopentene	–	–	0.27	0.24	0.21	–	–	831	832	831
4.467	nf	2,5,5-Trimethyl-1,3-cyclopentadiene	–	–	0.13	–	–	–	–	840	–	–
4.520	nf	1-Propyl-1-cyclopentene	–	–	0.16	0.15	0.12	–	–	843	843	843
4.593	841.0	4-Octyne	–	0.13	0.13	0.14	0.15	–	847	846	847	847
4.660	841.4	(3Z,5Z)-3,5-Octadiene	0.33	0.51	0.53	0.55	0.63	850	850	849	850	850
4.760	nf	1,3-Cyclooctadiene	–	–	0.11	0.12	0.12	–	–	854	854	855
4.840	857.2	Ethylbenzene	0.25	0.40	1.07	0.78	1.41	857	858	858	859	859
4.947	nf	1,2-Dimethylcyclohexene	–	0.16	0.19	0.21	0.23	–	864	864	864	864
5.033	868.0	1,3-dimethyl-benzene	–	0.48	0.77	0.84	1.12	–	868	868	868	868
5.213	879.0	Pentanoic acid	0.20	0.29	–	0.15	0.18	877	877	–	877	877

Table S1 (Continued)

Retention Time (min)	Retention Index (NIST)	Compound Name	Relative Area (%)					Retention Index				
			1A	2A	3A	4A	5A	1A	2A	3A	4A	5A
5.287	877.8	1,4-Dimethylbenzene	–	0.41	–	0.09	0.09	–	880	–	881	881
5.347	nf	1,3-dimethylene-2-methylcyclopentane	–	0.32	–	0.25	0.22	–	884	–	885	884
5.480	890.2	1-Nonene	0.97	1.98	0.83	1.55	1.50	890	891	890	891	891
5.527	891.2	1,2-dimethylbenzene	0.15	0.34	1.01	0.71	1.55	893	893	892	893	893
5.573	895.0	cis-4-Nonene	–	–	0.20	0.18	0.15	–	–	895	895	895
5.620	897.0	cis-3-Nonene	0.19	0.36	0.41	0.42	0.43	897	897	897	897	897
5.673	900.0	Nonane	0.35	0.87	1.25	1.02	1.20	900	900	900	900	900
5.733	nf	cis-Cyclooctene	1.34	0.88	0.41	0.49	0.69	902	903	902	903	903
5.800	909.0	trans-2-Nonene	–	0.28	0.44	0.43	0.53	–	906	905	906	906
5.893	nf	3-Allylcyclohexene	–	0.12	–	–	0.21	–	909	–	–	909
5.987	911.0	cis-2-Nonene	–	0.16	0.22	0.26	0.32	–	913	913	913	913
6.180	nf	1-Butylcyclopentene	–	0.14	0.11	0.15	0.13	–	921	921	921	921
6.280	918.0	1,3-Nonadiene	0.27	0.54	0.17	0.34	0.34	925	925	925	925	925
6.433	929.1	Propylcyclohexane	–	–	0.08	0.10	0.08	–	–	931	931	931
6.487	nf	Bicyclo(3.3.1)non-2-ene	–	0.16	–	0.20	0.11	–	933	–	934	934
6.633	nf	(1H)-3a,4,5,6,7,7a-Hexahydroindene	–	–	–	0.16	–	–	–	–	939	–
6.787	947.7	trans-Hexahydroindan	0.19	0.42	0.28	0.40	0.36	946	946	945	946	946
6.847	nf	cis-1,2-Diethylidenecyclopentane	–	0.28	0.11	0.18	0.15	–	948	948	948	948
6.900	nf	trans-1-Butenylcyclopentane	–	–	0.10	0.09	0.10	–	–	950	950	950
6.947	nf	3-Nonyne	–	0.13	–	–	–	–	952	–	–	–
6.973	952.3	Propylbenzene	–	0.21	0.67	0.48	0.77	–	953	953	953	953
7.013	nf	(2E,4E)-2,4-Nonadiene	–	0.20	0.13	0.14	0.17	–	955	955	955	955
7.087	nf	Ethylidenecycloheptane	–	0.12	0.08	0.12	0.10	–	958	958	958	958
7.160	960.8	1-ethyl-3-methyl-benzene	–	0.18	0.33	0.38	0.48	–	962	961	961	961
7.233	964.9	1,3,5-trimethyl-benzene	–	–	0.16	0.16	0.23	–	–	964	964	964

Table S1 (Continued)

Retention Time (min)	Retention Index (NIST)	Compound Name	Relative Area (%)					Retention Index				
			1A	2A	3A	4A	5A	1A	2A	3A	4A	5A
7.587	979.7	1-ethyl-2-methyl-benzene	–	–	0.66	0.51	0.89	–	–	978	978	978
7.640	981.0	Hexanoic acid	–	1.08	–	0.07	–	–	980	–	980	–
7.707	985.0	1-Methyl-1-phenylethylene	–	0.17	–	0.13	0.09	–	983	–	983	983
7.780	986.0	3-Octanone	–	–	–	0.11	–	–	–	–	986	–
7.900	990.7	1-Decene	0.96	1.97	0.88	1.72	1.56	991	991	991	991	991
7.987	993.9	trans-4-Decene	–	–	0.49	0.58	0.59	–	–	994	994	994
8.033	995.0	cis-3-Decene	–	0.46	0.46	0.51	0.51	–	996	996	997	996
8.127	1000.0	Decane	0.25	0.69	0.83	0.86	0.91	1000	1001	1000	1000	1000
8.260	1002.0	trans-2-Decene	–	0.22	0.40	0.39	0.32	–	1006	1005	1005	1006
8.460	1013.0	cis-2-Decene	–	0.12	0.23	0.22	0.25	–	1014	1013	1013	1013
8.533	nf	1-Butyl-1-cyclohexene	0.23	0.26	–	0.07	–	1016	1016	–	1016	–
8.667	1022.7	1,2,3-Trimethylbenzene	–	–	–	0.20	0.25	–	–	–	1021	1021
8.767	nf	cis-cyclodecene	–	0.22	–	0.19	0.18	–	1025	–	1025	1025
8.820	928.0	3-Phenyl-1-propene	–	0.11	0.10	0.08	0.13	–	1028	1027	1028	1028
9.007	1035.5	Indane	–	0.13	0.37	0.46	0.46	–	1035	1035	1035	1035
9.260	1045.0	Indene	–	–	0.30	0.19	0.28	–	–	1045	1045	1045
9.413	1052.0	1-Methyl-3-propylbenzene	–	0.22	0.14	0.17	0.19	–	1051	1051	1051	1051
9.547	1055.9	butyl-benzene	0.21	0.33	0.83	0.72	0.92	1056	1056	1056	1056	1056
9.620	1056.7	1,2-diethyl-benzene	–	–	0.16	0.09	0.16	–	–	1059	1059	1058
9.780	1059.1	1-methyl-4-propyl-benzene	–	–	0.48	0.29	0.47	–	–	1065	1065	1065
10.080	1076.0	Heptanoic acid	1.13	1.95	–	0.70	0.20	1077	1078	–	1079	1079
10.207	1079.3	1-Methylindan	–	–	0.13	0.30	0.32	–	–	1082	1082	1082
10.247	nf	1,10-Undecadiene	–	0.27	–	–	–	–	1083	–	–	–
10.300	1085.8	1,2-Dimethyl-3-ethylbenzene	–	–	0.26	0.53	0.53	–	–	1085	1086	1086
10.453	1091.4	1-Undecene	1.10	1.97	1.25	2.16	2.05	1091	1092	1091	1091	1091

Table S1 (Continued)

Retention Time (min)	Retention Index (NIST)	Compound Name	Relative Area (%)					Retention Index				
			1A	2A	3A	4A	5A	1A	2A	3A	4A	5A
10.573	nf	cis-4-undecene	–	–	0.36	0.48	0.45	–	–	1096	1096	1096
10.673	1100.0	Undecane	0.27	0.73	0.76	0.93	0.98	1100	1100	1100	1100	1100
10.793	1104.0	trans-2-Undecene	0.64	1.21	0.69	1.11	1.13	1105	1105	1105	1105	1105
11.007	1114.0	cis-2-Undecene	0.19	0.42	0.39	0.55	0.55	1113	1114	1113	1114	1113
11.100	nf	3-Isopropenyl-1-cyclooctene	–	0.21	–	0.18	0.16	–	1117	–	1117	1117
11.320	nf	(5E,7E)-5,7-Dodecadiene	0.67	1.09	0.41	0.78	0.75	1126	1127	1126	1126	1126
11.427	nf	1-Isopropenyl-3-propenylcyclopentane	–	0.40	–	0.21	0.30	–	1131	–	1130	1130
11.653	nf	Naphthalene,1,2,3,4,4a,5,6,8a-octahydro-4a-methyl-trans-	0.19	–	–	–	–	1139	–	–	–	–
11.680	1141.6	4-Methylindane	–	0.32	0.30	0.44	0.42	–	1140	1140	1140	1140
11.727	1143.0	1,3-diethyl-5-methyl-benzene	0.20	0.28	0.39	0.32	0.52	1142	1143	1142	1143	1142
11.907	nf	2-methyl-indene	0.34	0.91	0.62	1.17	1.22	1150	1150	1150	1150	1150
12.027	nf	1-(2-methylphenyl)-2-propanone	–	0.15	–	0.53	0.44	–	1155	–	1154	1155
12.107	1157.0	Pentylbenzene	0.63	0.90	1.88	2.36	2.87	1158	1158	1158	1158	1158
12.153	nf	6-Butyl-1,4-cycloheptadiene	0.48	0.62	–	–	–	1159	1159	–	–	–
12.240	1163.0	Tetrahydronaphthalene	–	–	–	0.23	0.25	–	–	–	1163	1163
12.320	nf	1-Isobutyl-4-methylbenzene	–	0.21	0.91	0.68	0.98	–	1166	1166	1166	1166
12.440	1170.0	Octanoic acid	0.28	0.79	–	0.18	–	1171	1172	–	1170	–
12.853	1187.0	Naphthalene	–	–	0.42	0.52	0.46	–	–	1187	1187	1187
12.960	1192.0	1-Dodecene	0.97	1.88	1.28	1.83	1.85	1192	1192	1192	1192	1192
13.060	nf	cis-4-Dodecene	–	–	0.34	0.39	0.33	–	–	1196	1196	1196
13.173	1200.0	Dodecane	0.23	0.64	0.83	0.91	0.90	1200	1201	1200	1200	1200
13.287	1205.0	trans-2-Dodecene	–	–	0.33	0.27	0.32	–	–	1205	1205	1205
13.500	1213.0	cis-2-Dodecene	–	–	0.26	0.17	0.22	–	–	1214	1214	1214
13.680	nf	Benzene, 1-methyl-2-(1-ethylpropyl)	0.25	0.22	0.09	0.15	0.17	1223	1223	1222	1222	1222

Table S1 (Continued)

Retention Time (min)	Retention Index (NIST)	Compound Name	Relative Area (%)					Retention Index				
			1A	2A	3A	4A	5A	1A	2A	3A	4A	5A
13.780	nf	2-Ethylindane	0.26	0.50	0.22	0.34	0.32	1226	1227	1226	1226	1226
13.953	1231.3	5-Ethylindan	–	0.12	0.22	0.22	0.25	–	1233	1233	1233	1233
14.120	nf	1,4-Dimethyl-2-isobutylbenzene	–	–	0.26	0.20	0.32	–	–	1240	1240	1240
14.527	nf	1H-Indene, 1,3-dimethyl-	–	0.24	0.20	0.18	0.24	–	1258	1257	1257	1257
14.613	1260.9	Hexylbenzene	–	0.65	1.04	0.78	1.08	–	1261	1261	1261	1261
14.733	nf	(1-methylpentyl)benzene	–	–	0.69	0.40	0.68	–	–	1266	1266	1266
14.753	1268.0	Nonanoic acid	0.26	0.73	–	0.07	0.18	1267	1268	–	1268	1268
14.847	nf	1,1-Dimethylindene	–	–	0.29	–	0.12	–	–	1271	–	1271
15.267	nf	(6E)-6-Tridecene	–	–	0.19	0.21	0.16	–	–	1288	1288	1288
15.353	1292.2	1-Tridecene	0.88	1.96	1.31	2.06	1.85	1292	1292	1292	1292	1292
15.433	nf	cis-4-tridecene	–	–	0.17	0.14	0.10	–	–	1295	1296	1296
15.547	1300.0	Tridecane	0.61	1.53	2.15	2.01	2.20	1300	1301	1300	1300	1300
15.653	1305.0	trans-2-tridecene	–	–	0.27	0.21	0.22	–	–	1305	1305	1305
15.867	1313.4	1-methyl-naphthalene	–	–	0.39	0.26	0.44	–	–	1314	1315	1315
15.973	nf	1-methyl-2-(2-methylpentyl)benzene	–	–	0.13	–	0.12	–	–	1319	–	1319
16.413	nf	(1-Ethylpentyl)benzene	–	–	0.14	0.29	0.14	–	–	1339	1339	1339
16.800	nf	7-phenyl-2-heptene	0.55	0.23	–	0.24	–	1355	1356	–	1355	–
16.993	1362.9	Heptylbenzene	–	–	0.65	0.51	0.51	–	–	1364	1364	1364
17.000	1368.2	Decanoic acid	0.62	0.79	–	–	–	1365	1364	–	–	–
17.073	nf	(1-methyl-hexyl)-benzene	–	–	0.30	0.11	0.21	–	–	1368	1368	1368
17.507	1391.0	trans-5-Tetradecene	–	–	0.18	0.28	0.18	–	–	1387	1387	1387
17.620	1392.2	1-Tetradecene	2.16	3.85	1.67	3.67	3.29	1392	1393	1392	1392	1392
17.680	1396.0	trans-7-tetradecene	–	–	0.17	0.23	0.17	–	–	1395	1395	1395
17.733	1396.1	2-Ethylnaphthalene	–	–	0.24	0.18	0.25	–	–	1397	1397	1397
17.793	1400.0	Tetradecane	0.50	0.89	1.09	0.87	0.89	1400	1400	1400	1400	1400

Table S1 (Continued)

Retention Time (min)	Retention Index (NIST)	Compound Name	Relative Area (%)					Retention Index				
			1A	2A	3A	4A	5A	1A	2A	3A	4A	5A
17.893	nf	trans-2-tetradecene	–	–	0.43	0.39	0.41	–	–	1405	1405	1405
18.107	nf	cis-2-tetradecene	–	–	0.32	0.39	0.40	–	–	1415	1415	1415
18.860	1451.0	1-Cyclopentylnonane	–	0.22	0.15	0.22	0.14	–	1451	1450	1451	1450
18.980	nf	Tetradecahydrobenzo[10]annulene	0.29	0.44	0.30	0.42	0.33	1455	1455	1456	1455	1455
19.093	1460.0	Undecanoic acid	–	0.82	–	0.22	–	–	1461	–	1463	–
19.240	1468.2	Octylbenzene	–	0.27	0.53	0.45	0.25	–	1469	1468	1468	1468
19.307	nf	2-phenyloctane	–	–	0.30	0.18	0.14	–	–	1471	1471	1471
19.389	nf	trans-4-Pentadecene	–	0.79	–	0.09	0.09	–	1475	–	1475	1475
19.507	nf	trans-5-Pentadecene	–	–	0.22	0.13	0.13	–	–	1481	1481	1481
19.553	nf	cis-5-Pentadecene	–	–	0.30	–	–	–	–	1483	–	–
19.573	nf	1,13-Tetradecadiene	0.18	0.44	–	0.30	0.32	1484	1484	–	1484	1484
19.627	nf	Cyclopentadecane	–	0.29	1.32	0.38	0.43	–	1487	1487	1487	1487
19.687	nf	cis-7-pentadecene	–	–	0.64	–	–	–	–	1489	–	–
19.753	1492.8	1-Pentadecene	0.92	3.75	2.80	2.06	2.14	1493	1493	1493	1493	1493
19.800	nf	trans-7-pentadecene	–	–	0.48	0.16	0.15	–	–	1495	1495	1495
19.920	1500.0	Pentadecane	2.22	3.18	13.02	2.84	3.78	1500	1500	1500	1500	1500
20.007	1507.0	trans-2-pentadecene	–	–	1.07	0.18	0.46	–	–	1505	1504	1504
20.227	1517.0	cis-2-pentadecene	–	–	0.87	0.21	0.44	–	–	1516	1516	1516
20.753	nf	1-heptyl-2,4-dimethylbenzene	–	–	0.15	–	–	–	–	1542	–	–
21.013	1556.4	Nonylcyclohexane	–	–	0.29	0.24	0.20	–	–	1555	1555	1555
21.367	1576.3	Nonylbenzene	0.23	–	0.57	0.91	0.92	1573	–	1573	1573	1573
21.387	nf	5-hexadecyne	–	1.01	–	–	–	–	1574	–	–	–
21.413	nf	Hexane, 2-phenyl-3-propyl-	–	–	0.46	–	–	–	–	1575	–	–
21.473	nf	8-Hexadecyne	–	0.61	–	0.36	0.26	–	1578	–	1578	1578
21.613	1587.0	2-Methyl-1-pentadecene	–	–	–	0.31	–	–	–	–	1585	–

Table S1 (Continued)

Retention Time (min)	Retention Index (NIST)	Compound Name	Relative Area (%)					Retention Index				
			1A	2A	3A	4A	5A	1A	2A	3A	4A	5A
21.687	1589.4	Fluorene	–	–	0.16	–	–	–	–	1589	–	–
21.773	1592.7	1-Hexadecene	1.31	2.26	1.07	1.69	1.32	1593	1593	1593	1593	1593
21.920	1600.0	Hexadecane	0.15	0.28	0.69	0.46	0.29	1600	1601	1600	1600	1600
22.007	1607.0	trans-2-hexadecene	–	–	0.40	0.29	0.26	–	–	1605	1605	1605
22.220	1618.0	cis-2-hexadecene	–	–	0.28	0.14	0.16	–	–	1616	1616	1616
23.013	1662.0	Tridecanoic acid	–	0.48	0.44	–	–	–	1659	1658	–	–
23.093	nf	Bi-1-cycloocten-1-yl	–	–	–	0.37	–	–	–	–	1662	–
23.140	nf	trans-6-Heptadecene	–	–	0.17	–	–	–	–	1665	–	–
23.247	1668.0	6(Z),9(E)-Heptadecadiene	0.48	1.08	0.50	0.63	0.55	1670	1671	1670	1670	1670
23.320	1675.7	(Z,Z,Z)-1,8,11,14-Heptadecatetraene	–	0.33	0.25	0.34	0.26	–	1674	1674	1674	1674
23.387	1677.7	Decylbenzene	1.48	2.06	2.16	1.52	1.53	1678	1678	1678	1678	1677
23.500	1680.0	trans-8-Heptadecene	0.61	1.04	2.07	0.81	0.81	1682	1682	1684	1683	1683
23.607	1692.0	trans-3-heptadecene	–	–	0.39	0.26	–	–	–	1689	1689	–
23.687	1692.0	1-Heptadecene	0.20	0.92	1.26	0.71	0.85	1693	1693	1693	1693	1693
23.773	nf	1-Decyl-3-methylbenzene	–	0.20	–	0.26	0.25	–	1698	–	1698	1698
23.820	1700.0	Heptadecane	0.26	0.48	2.81	0.40	0.54	1700	1700	1700	1700	1700
23.913	nf	1-Decyl-4-methylbenzene	–	0.35	1.18	0.38	0.52	–	1705	1706	1705	1705
24.000	1712.7	2-Methylfluorene	–	–	0.19	–	0.13	–	–	1710	–	1710
24.053	nf	1-methyl-4-(4-methylnonyl)benzene	–	–	0.12	–	–	–	–	1713	–	–
24.127	nf	1-Decyl-2-methylbenzene	–	0.29	0.83	0.32	0.39	–	1717	1717	1717	1717
24.267	nf	(6E,8E)-6,8-heptadecadiene	–	0.12	0.43	–	0.21	–	1725	1725	–	1725
24.327	nf	(6E,9E)-6,9-Heptadecadiene	0.32	0.41	0.32	0.21	0.30	1728	1728	1728	1728	1728
24.413	nf	1-(1-Decen-2-yl)-4-methylbenzene	–	–	0.21	–	–	–	–	1733	–	–
24.500	nf	1,16-Heptadecadiene	–	–	0.23	–	0.13	–	–	1738	–	1738
24.600	nf	1,8-Heptadecadiene	–	–	0.33	–	–	–	–	1744	–	–

Table S1 (Continued)

Retention Time (min)	Retention Index (NIST)	Compound Name	Relative Area (%)					Retention Index				
			1A	2A	3A	4A	5A	1A	2A	3A	4A	5A
24.687	nf	2-phenyl-2-methyldecane	–	–	0.41	–	–	–	–	1748	–	–
24.813	nf	6-cis,9-trans-Heptadecadiene	–	–	0.19	–	–	–	–	1755	–	–
24.887	1759.0	Tetradecanoic acid	0.40	0.32	0.65	0.21	0.21	1759	1759	1760	1760	1759
24.920	nf	(Z)6,(Z)9-Pentadecadien-1-ol	0.30	0.36	–	0.20	0.17	1761	1762	–	1762	1762
24.993	nf	1,4-dimethyl-2-(non-1-en-2-yl)benzene	–	–	0.28	0.08	0.08	–	–	1765	1766	1766
25.180	nf	1-methyl-4-(3-propylheptyl)benzene	0.35	0.51	0.33	0.39	0.37	1775	1775	1776	1775	1775
25.273	1779.8	Undecylbenzene	–	–	–	0.55	–	–	–	–	1781	–
25.313	nf	2-phenylundecane	–	–	1.12	–	0.54	–	–	1783	–	1784
25.413	1789.2	Phenanthrene	–	–	0.24	–	0.14	–	–	1789	–	1789
25.507	1793.5	1-Octadecene	0.16	0.34	0.27	0.30	0.27	1794	1794	1794	1794	1794
25.613	1800.0	Octadecane	–	0.16	0.34	0.19	0.19	–	1800	1800	1799	1799
25.920	1818.0	Hexadecanal	–	0.72	0.30	0.25	0.28	–	1818	1818	1818	1818
26.640	1862.0	di-2-methylpropyl phthalate	–	–	–	0.59	–	–	–	–	1860	–
27.093	nf	1-Dodecylbenzene	–	0.19	0.31	0.19	0.23	–	1886	1886	1886	1886
27.247	1895.0	1-Nonadecene	–	0.33	0.22	0.23	0.25	–	1894	1895	1894	1894
27.347	1901.0	2-Heptadecanone	0.76	3.41	1.32	3.44	2.21	1901	1901	1901	1901	1901
28.380	1962.0	Hexadecanoic acid	19.46	3.89	–	1.35	0.72	1964	1961	–	1960	1959
28.920	nf	3-octadecanone	–	0.52	0.14	0.48	0.31	–	1997	1997	1997	1997
30.033	nf	1-Methylpyrene	0.58	0.63	0.25	0.44	0.39	2269	2270	2269	2269	2269
30.080	2068.4	Fluoranthene	–	0.19	0.13	0.15	0.15	–	2071	2071	2071	2071
30.173	2077.0	(Z,Z)-2,13-octadecadienol	1.40	1.95	–	–	–	2077	2077	–	–	–
30.293	2082.0	Linoleic acid, methyl ester	1.41	2.13	0.79	1.77	1.51	2085	2085	2085	2085	2085
31.227	2145.8	Linoleic acid	28.22	–	–	–	–	2146	–	–	–	–
31.467	2162.0	Octadecanoic acid	2.16	–	–	–	–	2162	–	–	–	–
32.040	nf	Pentadecylbenzene	–	–	0.14	0.12	0.10	–	–	2200	2200	2200

Table S1 (Continued)

Retention Time (min)	Retention Index (NIST)	Compound Name	Relative Area (%)					Retention Index				
			1A	2A	3A	4A	5A	1A	2A	3A	4A	5A
32.787	nf	2-cis,cis-9,12-Octadecadienyloxyethanol	–	0.73	–	0.11	0.11	–	2252	–	2252	2252
32.880	nf	2-cis,trans-9,12-Octadecadienyloxyethanol	1.87	1.06	0.27	0.51	0.49	2259	2259	2259	2259	2259
45.300	3304.2	16-Hentriacontanone	0.27	1.22	0.27	0.78	0.46	3295	3295	3295	3295	3295

nf = not found.

Table S2. Absolute area of some ions detected via ESI(-)-FTMS. **1A** = WCSO, **2A** = BOWCSO, **3A** = BOα-Al, **4A** = BOMoCo, **5A** = BOγ-Al and **6A** = BOMoNi.

**Theoretical Exact Mass $[M-H]^-$**	**Elemental Composition**	**Absolute Area**					
		**1A**	**2A**	**3A**	**4A**	**5A**	**6A**
255.23295[b]	$C_{16}H_{31}O_2$	$1.5 \times 10^{+6}$	$1.4 \times 10^{+8}$	$1.3 \times 10^{+8}$	$3.5 \times 10^{+7}$	$1.3 \times 10^{+8}$	$3.8 \times 10^{+7}$
279.23295[c]	$C_{18}H_{31}O_2$	$1.8 \times 10^{+6}$	$1.6 \times 10^{+8}$	$2.3 \times 10^{+7}$	$5.4 \times 10^{+6}$	$2.9 \times 10^{+7}$	$1.2 \times 10^{+7}$
281.24860	$C_{18}H_{33}O_2$	$5.5 \times 10^{+5}$	$7.5 \times 10^{+7}$	$3.1 \times 10^{+7}$	$9.5 \times 10^{+6}$	$4.0 \times 10^{+7}$	$1.4 \times 10^{+7}$
283.26425	$C_{18}H_{35}O_2$	$8.0 \times 10^{+5}$	$1.0 \times 10^{+7}$	$1.2 \times 10^{+7}$	$4.9 \times 10^{+6}$	$9.3 \times 10^{+6}$	$5.5 \times 10^{+5}$
511.47318	$C_{32}H_{63}O_4$	-	$1.6 \times 10^{+7}$	$9.0 \times 10^{+6}$	$6.6 \times 10^{+5}$	$7.7 \times 10^{+6}$	$2.9 \times 10^{+6}$
535.47318	$C_{34}H_{63}O_4$	-	$3.4 \times 10^{+7}$	$3.4 \times 10^{+6}$	$2.6 \times 10^{+5}$	$3.8 \times 10^{+6}$	$1.9 \times 10^{+6}$
559.47318	$C_{36}H_{63}O_4$	-	$2.0 \times 10^{+7}$	$5.7 \times 10^{+5}$	$6.0 \times 10^{+4}$	$6.9 \times 10^{+5}$	$4.0 \times 10^{+5}$
561.48883	$C_{36}H_{65}O_4$	-	$1.8 \times 10^{+7}$	$9.8 \times 10^{+5}$	$7.9 \times 10^{+4}$	$1.3 \times 10^{+6}$	$6.5 \times 10^{+5}$

[b] base peak for all the catalytic bio-oils (BOα-Al, BOMoCo, BOγ-Al and BOMoNi);
[c] base peak for WCOS and BOWCSO.

Table S3. Absolute area of some ions detected via ESI(+)-FTMS. **1A** = WCSO, **2A** = BOWCSO, **3A** = BOα-Al, **4A** = BOMoCo, **5A** = BOγ-Al and **6A** = BOMoNi.

**Theoretical Exact Mass [M + Na]+**	**DAG or TAG**	**Elemental Composition**	**Absolute Area**					
			**1A**	**2A**	**3A**	**4A**	**5A**	**6A**
285.21889	-	$C_{18}H_{30}ONa$	-	$2.3 \times 10^{+6}$	$3.3 \times 10^{+6}$	$2.6 \times 10^{+6}$	$3.5 \times 10^{+6}$	$1.9 \times 10^{+6}$
301.14103[c]	-	$C_{16}H_{22}O_4Na$	$5.5 \times 10^{+5}$	$2.6 \times 10^{+5}$	$5.1 \times 10^{+5}$	$6.5 \times 10^{+5}$	$3.1 \times 10^{+7}$	$4.1 \times 10^{+5}$
301.25019[d]	-	$C_{19}H_{34}ONa$	-	$1.7 \times 10^{+6}$	$6.9 \times 10^{+6}$	$4.1 \times 10^{+6}$	$7.4 \times 10^{+6}$	$3.8 \times 10^{+6}$
335.25567	-	$C_{19}H_{36}O_3Na$	$3.0 \times 10^{+4}$	$1.7 \times 10^{+7}$	$6.7 \times 10^{+5}$	$4.6 \times 10^{+5}$	$9.2 \times 10^{+5}$	$7.5 \times 10^{+5}$
341.24510	-	$C_{21}H_{34}O_2Na$	-	$7.5 \times 10^{+6}$	$6.2 \times 10^{+6}$	$2.5 \times 10^{+6}$	$5.9 \times 10^{+6}$	$3.1 \times 10^{+6}$
359.25567[e]	-	$C_{21}H_{36}O_3Na$	$4.9 \times 10^{+3}$	$2.6 \times 10^{+7}$	$6.2 \times 10^{+5}$	$1.9 \times 10^{+5}$	$6.2 \times 10^{+5}$	$7.2 \times 10^{+5}$
381.29753[f]	-	$C_{21}H_{42}O_4Na$	$1.1 \times 10^{+6}$	$4.1 \times 10^{+5}$	$1.3 \times 10^{+7}$	$9.4 \times 10^{+5}$	$7.5 \times 10^{+4}$	-
615.49590	PL[a]	$C_{37}H_{68}O_5Na$	$2.5 \times 10^{+6}$	$7.3 \times 10^{+5}$	$1.9 \times 10^{+4}$	$4.6 \times 10^{+3}$	-	$4.6 \times 10^{+3}$
617.51155	PO[a]	$C_{37}H_{70}O_5Na$	$4.4 \times 10^{+5}$	$2.0 \times 10^{+5}$	$2.0 \times 10^{+3}$	$1.6 \times 10^{+3}$	-	-
639.49590	LL[a]	$C_{39}H_{68}O_5Na$	$2.9 \times 10^{+6}$	$5.0 \times 10^{+5}$	$3.1 \times 10^{+4}$	$7.6 \times 10^{+3}$	$2.0 \times 10^{+4}$	$3.8 \times 10^{+3}$
641.51155	OL[a]	$C_{39}H_{70}O_5Na$	$1.2 \times 10^{+6}$	-	$3.2 \times 10^{+4}$	$4.2 \times 10^{+3}$	$8.7 \times 10^{+3}$	$2.9 \times 10^{+3}$
643.52720	OO[a]	$C_{39}H_{72}O_5Na$	$3.6 \times 10^{+5}$	-	$3.6 \times 10^{+4}$	$2.9 \times 10^{+3}$	$5.5 \times 10^{+3}$	$5.0 \times 10^{+2}$
825.69079	PoPoP[b]	$C_{51}H_{94}O_6Na$	$9.3 \times 10^{+5}$	-	-	-	-	-
827.70644	PoPP[b]	$C_{51}H_{96}O_6Na$	$2.0 \times 10^{+5}$	-	-	-	-	-
829.72209	PPP[b]	$C_{51}H_{98}O_6Na$	$1.3 \times 10^{+5}$	-	-	-	-	-
849.69426	PoPoL[b]	$C_{53}H_{94}O_6Na$	$1.1 \times 10^{+6}$	$4.1 \times 10^{+4}$	-	-	-	-
851.70991	PPLn[b]	$C_{53}H_{96}O_6Na$	$1.0 \times 10^{+6}$	$4.3 \times 10^{+4}$	-	-	-	-
853.72556	PPL[b]	$C_{53}H_{98}O_6Na$	$1.1 \times 10^{+7}$	$2.6 \times 10^{+5}$	-	-	-	-
875.70991	PoLL[b]	$C_{55}H_{96}O_6Na$	$6.7 \times 10^{+5}$	$2.7 \times 10^{+4}$	-	-	-	-
877.72556[g]	PLL[b]	$C_{55}H_{98}O_6Na$	$2.7 \times 10^{+7}$	$3.0 \times 10^{+5}$	-	-	-	-
879.74121	PLO[b]	$C_{55}H_{100}O_6Na$	-	$2.1 \times 10^{+5}$	-	-	-	-
881.75686	POO[b]	$C_{55}H_{102}O_6Na$	-	$7.4 \times 10^{+4}$	-	-	-	-
901.72556	LLL[b]	$C_{57}H_{98}O_6Na$	$1.6 \times 10^{+7}$	$1.0 \times 10^{+5}$	-	-	-	-
903.74121	LLO[b]	$C_{57}H_{100}O_6Na$	$1.2 \times 10^{+7}$	-	-	-	-	-
905.75686	LOO[b]	$C_{57}H_{102}O_6Na$	$5.0 \times 10^{+6}$	$6.7 \times 10^{+4}$	-	-	-	-
907.77251	OOO[b]	$C_{57}H_{104}O_6Na$	$1.3 \times 10^{+6}$	$1.3 \times 10^{+4}$	-	-	-	-
909.78816	OOS[b]	$C_{57}H_{106}O_6Na$	$3.1 \times 10^{+5}$	$4.1 \times 10^{+3}$	-	-	-	-

P = palmitic acid, Po = palmitoleic acid, S = stearic acid, O = oleic acid, L = linoleic acid and Ln = linolenic acid;
[a] DAG = diacylglycerols;
[b] TAG = triacylglycerols;
[c] base peak for BOγ-Al;
[d] base peak for BOMoCo and BOMoNi;
[e] base peak for BOWCSO;
[f] base peak for BOα-Al;
[g] base peak for WCS
